# Neutrophil extracellular traps contribute to immunothrombosis formation via the STING pathway in sepsis-associated lung injury

**DOI:** 10.1038/s41420-023-01614-8

**Published:** 2023-08-25

**Authors:** Shuainan Zhu, Ying Yu, Mengdi Qu, Zhiyun Qiu, Hao Zhang, Changhong Miao, Kefang Guo

**Affiliations:** 1 Department of Anesthesiology, Zhongshan Hospital, Fudan University, Shanghai, China; 2 Shanghai Key Laboratory of Perioperative Stress and Protection, Shanghai, China

**Keywords:** Cell death, Respiratory tract diseases

## Abstract

Neutrophil extracellular traps (NETs) are involved in the activation and dysfunction of multiple overlapping and interacting pathways, including the immune response to injury, inflammation, and coagulation, which contribute to the pathogenesis of sepsis-induced acute lung injury (SI-ALI). However, how NETs mediate the relationship between inflammation and coagulation has not been fully clarified. Here, we found that NETs, through stimulator of interferon genes (STING) activation, induced endothelial cell damage with abundant production of tissue factor (TF), which magnified the dysregulation between inflammatory and coagulant responses and resulted in poor prognosis of SI-ALI model mice. Disruption of NETs and inhibition of STING improved the outcomes of septic mice and reduced the inflammatory response and coagulation. Furthermore, Toll-like receptor 2 (TLR2) on the surface of endothelial cells was involved in the interaction between NETs and the STING pathway. Collectively, these findings demonstrate that NETs activate the coagulant cascade in endothelial cells in a STING-dependent manner in the development of SI-ALI.

## Introduction

Sepsis is an extreme host response to infection that causes life-threatening organ damage and remains a major cause of death worldwide [1, 2]. Current practices in sepsis treatment focus on antibiotics, hemodynamic interventions, mediator modulation, mechanical ventilation, and organ support when required [3, 4]. The mortality of 30-day septic shock was 34.7%, and that of 90-day septic shock reached 38.5% [5]. Even for survivors, long-term quality of life is affected by both mental and physical impairment. Furthermore, in light of the COVID-19 pandemic, the number of septic patients increased sharply, and improving sepsis management is critical [6]. Acute lung injury (ALI) occurs in almost 50% of septic patients, as the lungs are particularly vulnerable to infection and account for the majority of sepsis-related deaths [7]. Sepsis-induced acute lung injury (SI-ALI) involves activation and dysfunction of multiple overlapping and interacting pathways, including immunity, inflammation, and coagulation, which result in widespread endothelial and epithelial damage [6, 8]. Recently, the concept that inflammation and coagulation are inextricably linked in the pathophysiology of ALI was emphasized, especially in the hyperinflammatory subphenotype [9]. The initial pro-inflammatory response triggers endothelial injury and coagulation cascades, which leads to the aggregation and activation of immune cells and the increased production of thrombin and inflammatory cytokines [10]. However, the precise mechanisms that link inflammation and coagulation have not been fully elucidated, and effective therapies for SI-ALI are limited.

Neutrophils migrate from the bloodstream and release a variety of pro-inflammatory mediators, initiating inflammation [11]. Neutrophil extracellular traps (NETs) composed of DNA scaffolds, histones, and granular proteases are also released during this process [12]. Current evidence shows that NETs induce a pro-inflammatory and pro-coagulant endothelial phenotype through the degradation of the anti-coagulation system and upregulation of tissue factor (TF) [13], suggesting that NETs are a bridge between inflammation and coagulation. We previously reported that TF-enriched NETs in sepsis caused immunothrombosis formation and worse outcomes of SI-ALI model mice [14]. As neutrophils were not the major source of TF, we wondered whether NETs induce TF expression through indirectly inflammation in SI-ALI model mice.

NET components can directly impair cell integrity or interact with cell-intrinsic immune pathways to affect cell fate [15]. The stimulator of interferon genes (STING), encoded by Tmem173, was originally identified as a key modulator in producing type I interferons (IFNs) and inflammatory cytokines during the DNA-driven immune response [16]. Robust activation of STING is present in the process of sepsis-driven inflammation, coagulation, and tissue damage [17–19]. Global depletion or conditional ablation of STING in myeloid cells mitigated lethal coagulation in septic mouse models. Mechanistically, STING mediates TF release through gasdermin D-dependent pyroptosis [20]. Apel et al. reported that cGAS-STING was a sensor of NETs and mediated immunity during infection, suggesting that cGAS-STING can act as a drug target for the treatment of NET-associated diseases [21]. However, whether the STING pathway affects the NET-induced imbalance between inflammation and coagulation in endothelial cells as an important intercellular communicator has not been clarified.

Here, we showed that the number of NETs was significantly elevated in septic patients and SI-ALI model mice, thus causing dysregulated inflammation and coagulation and leading to poor outcomes. We observed that NETs induce endothelial damage and TF expression through activation of the cGAS-STING pathway, which requires a TLR2 sensor on the surface of endothelial cells. Overall, our findings suggest that STING functions as a mediator of NET-driven inflammation and coagulation in sepsis, opening the possibility of exploring drugs that target STING for the treatment of SI-ALI.

## Results

### NET formation correlated with SI-ALI and coagulation cascades

**Fig. 1:**
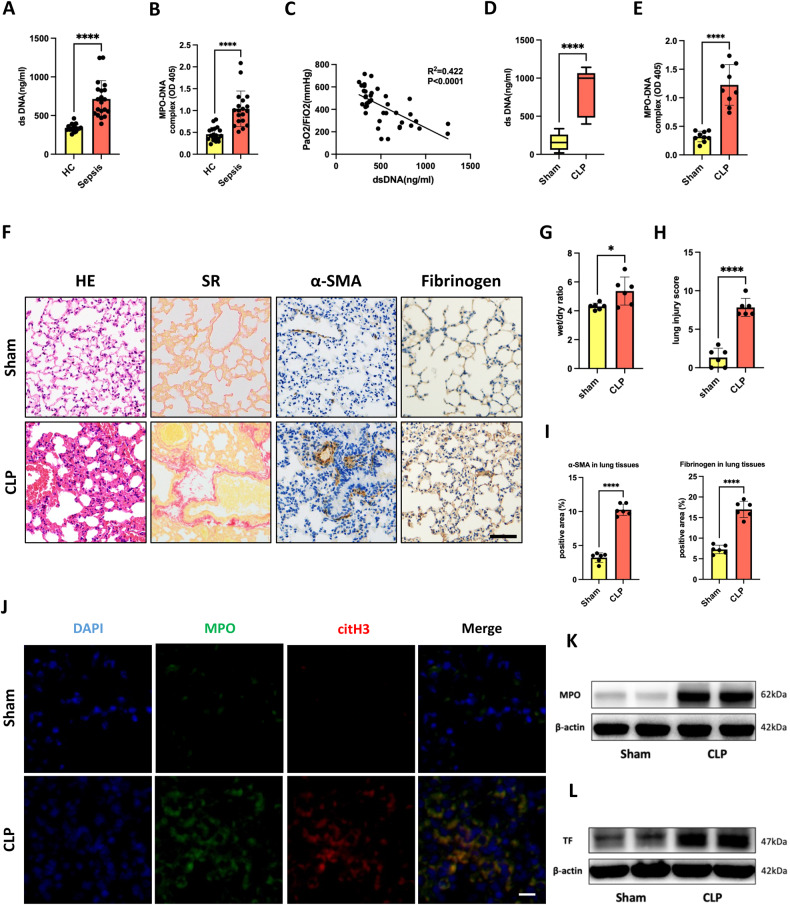
NET formation correlated with SI-ALI and coagulation cascades. **A** Levels of dsDNA in the serum of septic patients (*n* = 20) and healthy controls (HCs) (*n* = 20). **B** Plasma MPO–DNA levels of septic patients (*n* = 20) and HCs (*n* = 20). **C** Correlation curves between dsDNA and PaO2/FiO2. **D** Plasma levels of dsDNA in septic mouse models (*n* = 9). **E** Plasma MPO–DNA levels in septic mouse models (*n* = 9). **F** Normal and septic lung sections were subjected to H&E staining, Sirius Red staining, α-SMA, and fibrinogen immunohistochemical analysis (*n* = 6; scale bar: 100 μm). **G** The lung wet/dry ratio (*n* = 6). **H** Lung injury was semi quantified according to H&E staining. The lung injury score was recorded (*n* = 6). **I** The proportions of the α-SMA-positive areas and fibrinogen-positive areas in lung tissues were calculated by Fiji/ImageJ (NIH) software (*n* = 6). **J** Representative immunofluorescence images of NETs in lung tissues from sham and sepsis model mice. Scale bar: 20 μm. **K** Western blot images of MPO expression in lung tissues. **L** Western blot images of TF expression in lung tissues. Each bar represents the mean ± SD. The comparison between the two groups was performed using unpaired t-tests (**A**, **B**, **D**, **E**, and **G**–**I**). *p < 0.05, **p < 0.01, ***p < 0.001.

To investigate the involvement of NETs in SI-ALI and coagulation cascades, we measured the levels of dsDNA and MPO-DNA, the well-established indicators of NET formation, in patients with sepsis and matched healthy controls. In line with our previous reports, the NET formation was significantly increased in septic patients (Fig. 1A, B) and positively correlated with lung injury (Fig. 1C). Compared with healthy controls, septic patients had obviously dysregulated coagulation cascades and presented delayed prothrombin time (PT), increased partial thromboplastin time (APTT), increased plasma fibrinogen (FIB) and D-dimer levels, and reduced platelet numbers (Supplementary Fig. 1A–E). Given that NETs participate in septic immune-thrombosis formation, we further conducted a correlation analysis between NET formation and coagulation markers. The positive correlation of serum dsDNA levels with PT, APTT, FIB, and D-dimer (Supplementary Fig. 1F–I) suggested that enhanced NETosis levels in the development of sepsis may modulate the dysregulated coagulation system. Moreover, the cecal ligation and puncture (CLP) mouse model is the most commonly used septic model to mimic the progression and characteristics of human sepsis. In line with what we observed in septic patients, CLP sepsis model mice exhibited severe ALI and increased circulating and lung NET formation (Fig. 1D–K, Supplementary Fig. 1J). In addition, the level of fibrinogen deposition and TF expression (the primary initiator of blood coagulation) in lung tissue was drastically increased (Fig. 1F, I, L), indicating coagulation activation. Overall, these findings suggested that NETs play a key role in the development of septic ALI and coagulation cascades.

### NET inhibition protected mice against sepsis-induced ALI and coagulation activation

**Fig. 2:**
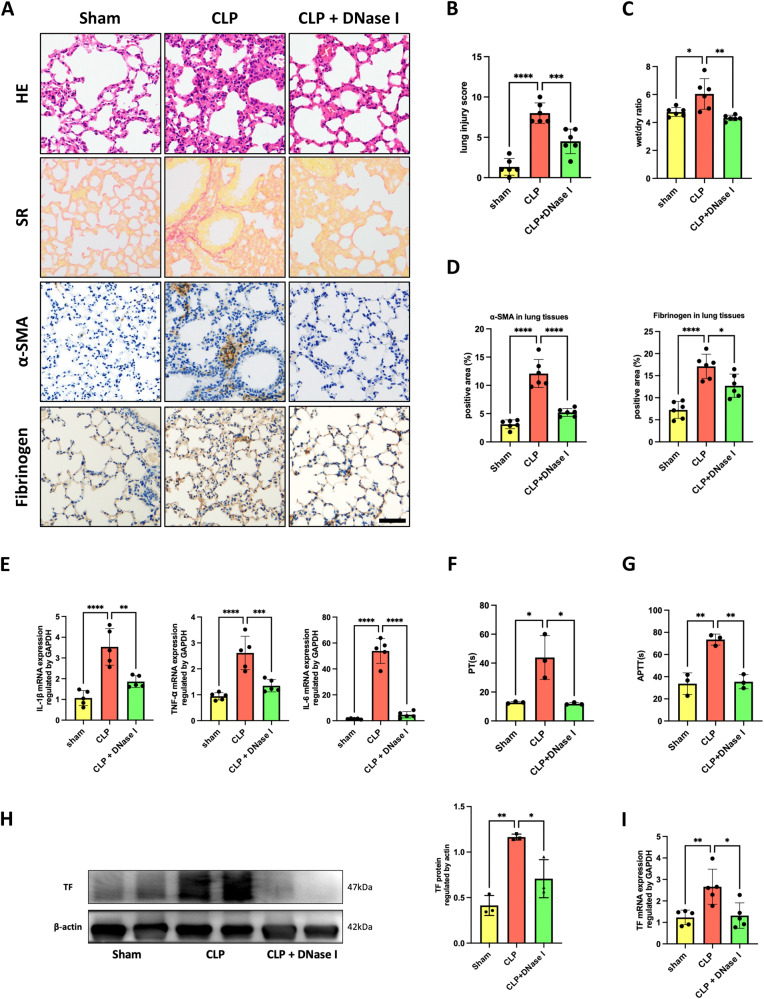
NET inhibition protected mice against SI-ALI and coagulation activation. Degradation of NETs using DNase I protects against lung injury and coagulation induced by CLP. **A** H&E staining, Sirius Red staining, α-SMA, and fibrinogen immunohistochemical analysis of the lung tissues. Scale bar: 100 μm. **B** Lung injury was semi quantified according to H&E staining. The lung injury score was recorded (*n* = 6). **C** The lung wet/dry ratio (*n* = 6). **D** The proportions of the α-SMA-positive areas and fibrinogen-positive areas in lung tissues were calculated by Fiji/ImageJ software (*n* = 6). **E** The mRNA levels of TNF-α, IL-1β, and IL-6 in murine lung tissues (*n* = 5). **F**, **G** PT and APTT were assayed from murine plasma (*n* = 3). **H** Western blot images of TF expression in lung tissues. **I** The mRNA level of TF in murine lung tissues (*n* = 5). Each bar represents the mean ± SD. Statistical analysis for three or more groups was carried out using 1-way ANOVA (**B**–**I**). *p < 0.05, **p < 0.01, ***p < 0.001.

To assess whether NETs mediate the inflammation and coagulation cascades during sepsis, we induced NET degradation with DNase I. Histopathological analysis of septic mouse lung sections showed alleviated hemorrhage and alveolar edema, with less leukocyte infiltration and alveolar thickness after treatment with DNase I (Fig. 2A, B). Consistently, lung fibrosis (Fig. 2A, D) and the wet-to-dry weight ratio were ameliorated (Fig. 2C), with reduced inflammatory cytokine production in the NET inhibition group (Fig. 2E). Furthermore, we observed less fibrinogen deposition (Fig. 2A, D) and decreased PT and APTT (Fig. 2F, G) and TF expression in the lung (Fig. 2H, I), all indicating that sepsis-induced coagulation activation was attenuated. These findings supported the important role of NETs in mediating SI-ALI and coagulation activation.

### NETs upregulated TF expression in endothelial cells

**Fig. 3:**
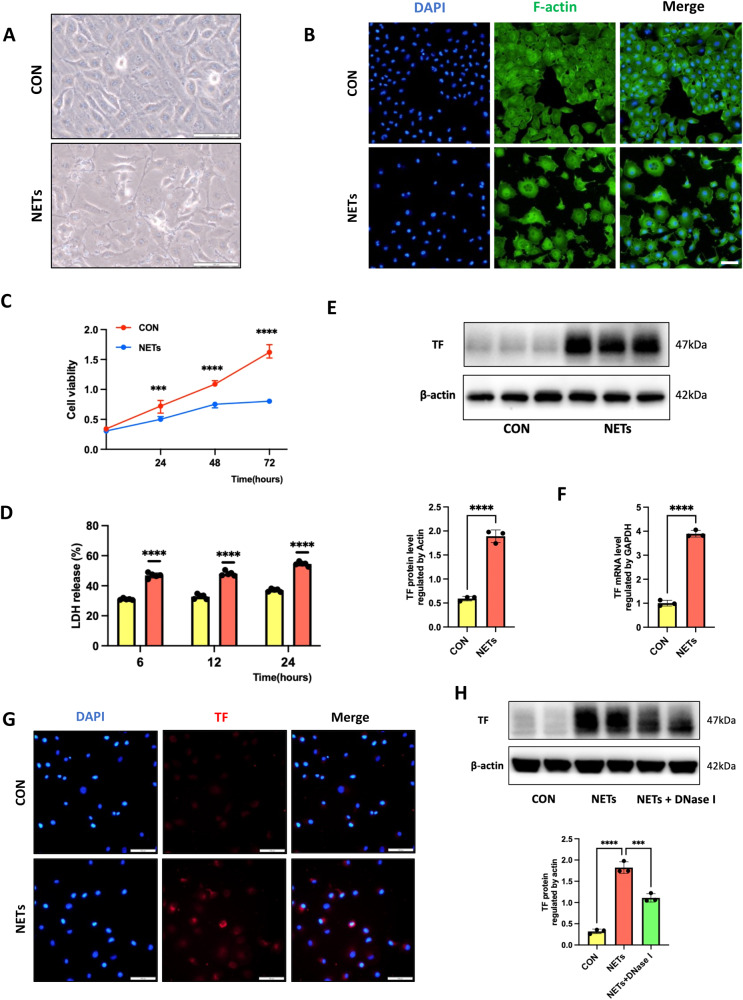
NETs upregulated TF expression in endothelial cells. **A** Representative images of control and NET-treated HUVECs. Scale bar: 100 μm. **B** Representative immunofluorescence staining of the cytoskeleton in control and NET-treated HUVECs. Scale bar: 100 μm. **C** The cell viability of HUVECs treated with NETs (*n* = 5). **D** The cellular supernatant LDH level was evaluated using a cytotoxicity detection LDH kit (*n* = 5). **E** Western blot images of TF expression in control and NET-treated HUVECs. **F** Relative mRNA levels of TF in control and NET-treated HUVECs (*n* = 3). **G** Representative images of immunofluorescence staining of TF in HUVECs. Scale bar: 100 μm. **H** Western blot images of TF expression in HUVECs with treatments. Each bar represents the mean ± SD. The comparison between the two groups was performed using unpaired t-tests (**C**–**F**). Statistical analysis for three or more groups was carried out using 1-way ANOVA (**H**). *p < 0.05, **p < 0.01, ***p < 0.001.

Since TF-enriched NET formation contributes to immunothrombosis in septic and acute respiratory distress syndrome (ARDS) patients, but the precise mechanisms of TF exposure have not been fully elucidated, we next investigated the source of TF. Endothelial cells are one of the common sources of TF, and they comprise the first barrier NETs need to pass through to reach the alveolar parenchyma. We hypothesize that NETs induce abnormal TF expression in endothelial cells in the process of exerting their defensive functions. To test this hypothesis, we treated human umbilical vein endothelial cells (HUVECs) with NETs (PMA-stimulated neutrophil media, PMA was used to stimulate NET formation) for 24 h, and the morphology and other related markers were evaluated. Compared with control cells, NET-treated HUVECs exhibited decreased cell viability when observed using optical microscopy (Fig. 3A), accompanied by marked destruction of the cytoskeleton (Fig. 3B). Cell viability and cytotoxicity were then measured using a CCK-8 assay and an LDH cytotoxicity assay, respectively. Treatment with NETs resulted in a significant decline in cell viability and increased LDH release in a time-dependent manner (Fig. 3C, D). We further found a significant alteration in the transcript levels of several key components of the coagulation cascade in HUVECs stimulated with NETs compared with control cells, including a marked upregulation of TF. Next, we measured the TF expression levels in cell lysates. NETs induced a nearly 4-fold change in TF expression in HUVECs, as well as increased TF staining in immunofluorescence images (Fig. 3E–G). Similar to the observation in mouse lung tissues, degradation of NETs using DNase I abrogated this effect (Fig. 3H). Collectively, these results supported that NETs enhanced TF expression in endothelial cells.

### NETs upregulated the expression of TF in endothelial cells through activation of the STING pathway

**Fig. 4:**
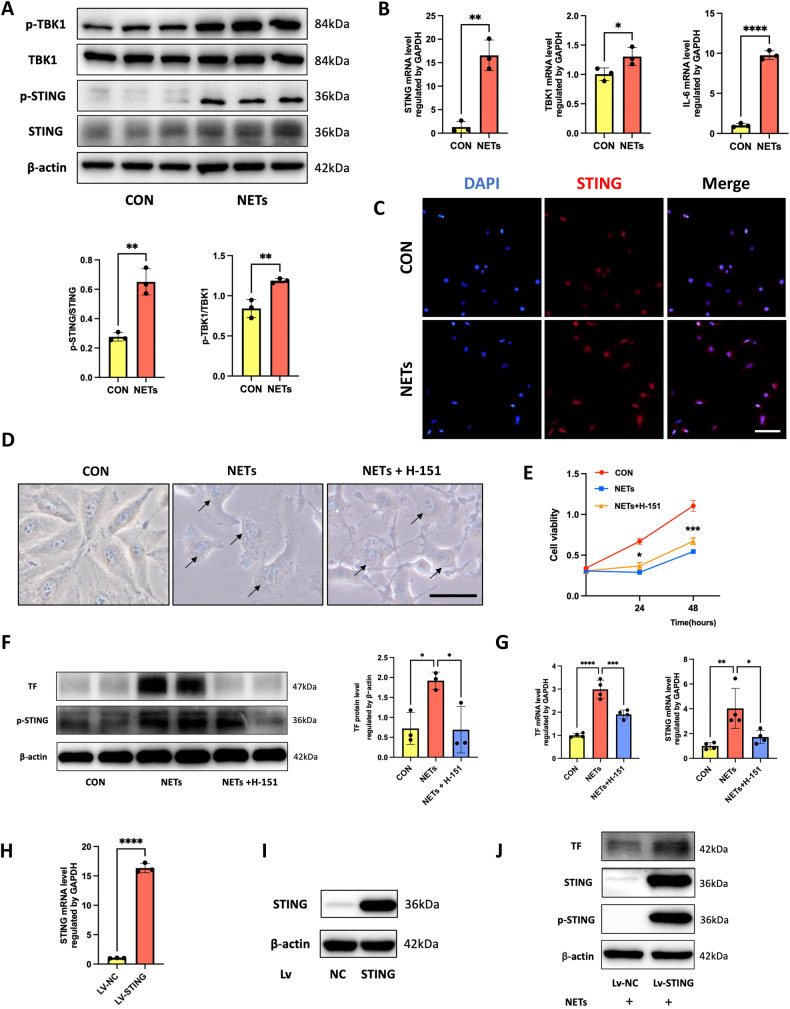
NETs upregulated TF expression in endothelial cells through activation of the STING pathway. **A** Western blot images of the STING activation in HUVECs. **B** Relative mRNA levels of the STING activation in HUVECs (*n* = 3). **C** Representative images of immunofluorescence staining of STING in HUVECs. Scale bar: 100 μm. **D** Representative optical images of control and NET-treated HUVECs with or without the STING inhibitor H-151. Scale bar: 100 μm. **E** The cell viability of HUVECs with different treatments using the CCK-8 assay (*n* = 4). **F** Western blot images of p-STING and TF expression in HUVECs with different treatments. **G** Relative mRNA levels of STING and TF in HUVECs with different treatments (*n* = 4). **H** Relative mRNA levels of STING in HUVECs transfected with LV-negative control (NC) or LV-STING (*n* = 3). **I** Western blot images of STING expression in HUVECs transfected with LV-NC or LV-STING. **J** Western blot images of STING and TF expression in NET-treated HUVECs transfected with LV-NC or LV-STING. Each bar represents the mean ± SD. The comparison between the two groups was performed using unpaired t-tests (**A**, **B**, and **H**). Statistical analysis for three or more groups was carried out using 1-way ANOVA (**E** and **G**). *p < 0.05, **p < 0.01, ***p < 0.001.

Previous studies have demonstrated that NETs interact with innate immune pathways, which led us to hypothesize that the innate immune sensor STING pathway contributes to NET-induced TF expression, as STING-dependent GSDMD activation has been shown to trigger excessive TF release in a sepsis mouse model [20]. To identify the intracellular signaling components that mediate the modulatory effects of NET-induced TF expression, we measured the levels of STING activation. Compared with control cells, the NET-treated cells showed increased phosphorylation of STING and TBK1, accompanied by upregulated TF expression (Fig. 4A). We then found that NETs increased STING, TBK1, and IL-6 transcript levels (Fig. 4B) and NET-treated cells exhibited prominent STING staining in immunofluorescence images (Fig. 4C), indicating that the STING pathway may contribute to NET-induced cell fate changes. Furthermore, pretreatment with the STING selective inhibitor H-151 (Med Chemical Express) before NET stimulation partially reversed cell morphology damage and cell viability reductions (Fig. 4D, E). We next measured TF and STING activation at both the protein and mRNA levels and observed that TF was downregulated upon STING inhibition (Fig. 4F, G). In contrast, lentivirus-mediated overexpression of STING, in which robust overexpression was validated in both mRNA and protein aspects (Fig. 4H, I), exhibited a much higher level of TF expression after stimulation with NETs (Fig. 4J). In summary, these results suggested that STING was a key mediator of NET-induced cell damage and TF expression.

### NETs promoted STING upregulation by activating TLR2 signaling

**Fig. 5:**
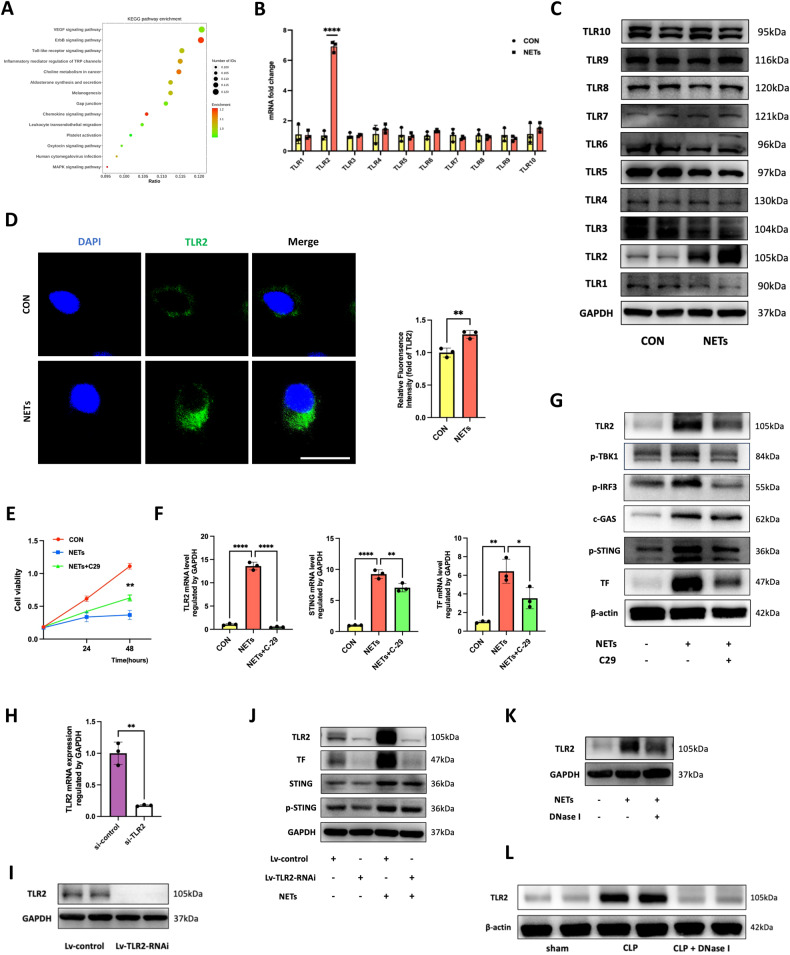
NETs promoted STING upregulation by activating TLR2 signaling. **A** KEGG analysis identified significantly altered pathways in control and NET-treated HUVECs. **B** Relative mRNA levels of TLRs in control and NET-treated HUVECs (*n* = 3). **C** Western blot images of TLRs expression in control and NET-treated HUVECs. **D** Representative images of immunofluorescence staining of TLR2 in control and NET-treated HUVECs. Scale bar: 30 μm. The expression of TLR2 was assessed by fluorescence intensity (*n* = 3). **E** The cell viability of HUVECs pretreated with C-29 (*n* = 3). **F** Relative mRNA levels of TLR2, STING, and TF in HUVECs pretreated with C-29 (*n* = 3). **G** Western blot images of STING pathway and TF expression in HUVECs pretreated with C-29. **H** Relative mRNA levels of STING in HUVECs transfected with LV-NC-RNAi or LV-TLR2-RNAi (*n* = 3). **I** Western blot images of TLR2 expression in HUVECs transfected with LV-NC-RNAi or LV-TLR2-RNAi. **J** Western blot was performed to analyze the levels of STING and TF expression in HUVECs transfected with LV-NC-RNAi or LV-TLR2-RNAi under stimulation of NETs. **K** Western blot images of TLR2 expression in HUVECs pretreated with DNase I. **L** Western blot images of TLR2 expression in murine lung tissues treated with DNase I. Each bar represents the mean ± SD. The comparison between the two groups was performed using unpaired t-tests (**B**, **D**, and **H**). Statistical analysis for three or more groups was carried out using 1-way ANOVA (**E** and **F**). *p < 0.05, **p < 0.01, ***p < 0.001.

Given that NETs triggered an innate immune and inflammatory response through the activation of damage-associated molecular patterns and that TLRs were implicated in various pathologies as important sensors of products of damaged tissues [22], as well as high affinity to ligands sourced from bacterial cell wall components or lipoproteins during infections, we wondered if TLRs play a role in NET-treated HUVECs. We reanalyzed our RNA-seq data and found that the Toll-like receptor pathway was upregulated in NET-stimulated HUVECs (Fig. 5A). Additionally, only the mRNA level of TLR2, among 10 TLR family members, was markedly increased, according to the screen (Fig. 5B). Further, we explored the protein level of TLR family in NET-induced HUVECs. Consistent with mRNA fold change, TLR2 protein expression was undetectable in control cells but significantly upregulated in NET-treated HUVECs (Fig. 5C). To observe TLR2 expression stimulated by NETs in HUVECs, we performed immunofluorescence analysis. Fluorescence intensity of TLR2 was increased under the stimulation of NETs and appeared more in cytosol (Fig. 5D). When we pretreated HUVECs with a known TLR2 antagonist (C29, Med Chemical Express) before NET stimulation, the NET-induced decline in cell viability was partially reversed (Fig. 5E), suggesting a potential role of TLR2 in NET-stimulated cell damage. Additionally, TLR2 inhibition alleviated NET-induced TF expression and activation of the STING pathway (Fig. 5F, G). To further verify the role of TLR2 in NETs sensing, we constructed Lentivirus-TLR2-RNAi to silence TLR2 in HUVECs, and the efficacy was assessed by qPCR and western blot (Fig. 5H, I). In response to NETs stimulation, the knockdown of TLR2 attenuated the damage of NETs to HUVECs with reduced TF and STING activation (Fig. 5J). These data further strengthened our hypothesis that NETs-induced TF upregulation was dependent on TLR2 signaling. Additionally, after adding DNase I to degrade NETs to the culture medium, TLR2 expression was significantly inhibited (Fig. 5K). In line with the in vitro experiments, TLR2 expression was much higher in the lung tissues of the CLP group than in those of the sham group, and adding DNase I to degrade NETs observably reversed TLR2 upregulation (Fig. 5L). Collectively, these findings demonstrated that NET-induced STING activation and TF expression were partially dependent on TLR2-mediated innate immune signaling.

### TLR2 deficiency ameliorated SI-ALI and coagulation activation in the CLP mouse model

**Fig. 6:**
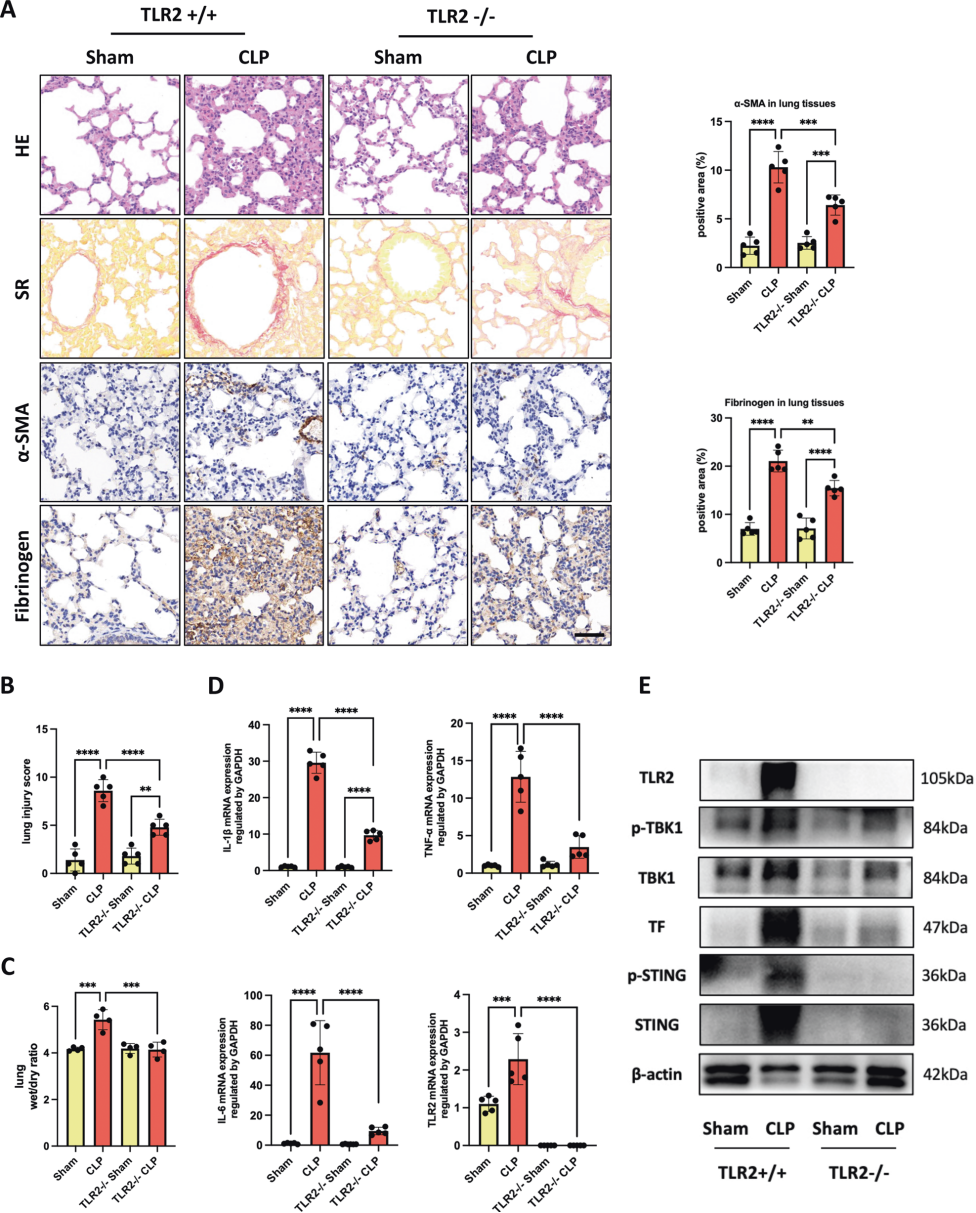
TLR2 deficiency ameliorated SI-ALI and coagulation activation in the CLP mouse model. **A** H&E staining, Sirius Red staining, α-SMA, and fibrinogen immunohistochemical analysis of the lung tissues (*n* = 5; Scale bar: 50 μm). The proportions of the α-SMA-positive areas and fibrinogen-positive areas were calculated by Fiji/ImageJ software (*n* = 5). **B** Lung injury was semi quantified according to H&E staining. The lung injury score was recorded (*n* = 5). **C** The lung wet/dry ratio (*n* = 4). **D** The mRNA levels of TNF-α, IL-1β, and IL-6 in murine lung tissues (*n* = 5). **E** Western blot images of STING pathway and TF expression in lung tissues. Statistical analysis for three or more groups was carried out using 1-way ANOVA (**A**–**D**). *p < 0.05, **p < 0.01, ***p < 0.001.

To investigate whether knockout of TLR2 could abolish NETs-induced proinflammatory and procoagulant effect in vivo, TLR2 knockout (TLR2−/−) mice were used to establish a CLP-induced ALI model. As expected, histopathological analysis of TLR2−/− septic mouse lung sections showed alleviated hemorrhage and alveolar edema, with less leukocyte infiltration and alveolar thickness compared with those in TLR2+/+ mice (Fig. 6A, B). Consistently, lung fibrosis (Fig. 6A) and the wet-to-dry weight ratio were ameliorated (Fig. 6C), with reduced inflammatory cytokine production in the TLR2−/− group (Fig. 6D). Similarly, NETs-mediated fibrinogen deposition (Fig. 6A) and TF expression in the lung (Fig. 6E) were also significantly reduced in TLR2−/− mice group, accompanied by reduced STING activation, all indicating that sepsis-induced coagulation activation was attenuated. In summary, these findings supported the important role of TLR2 in mediating interaction between NETs and lung parenchymal cells, thereby contributing to SI-ALI and coagulation activation.

### Interruption of the STING pathway alleviated NET-induced TF expression in a CLP mouse model

**Fig. 7:**
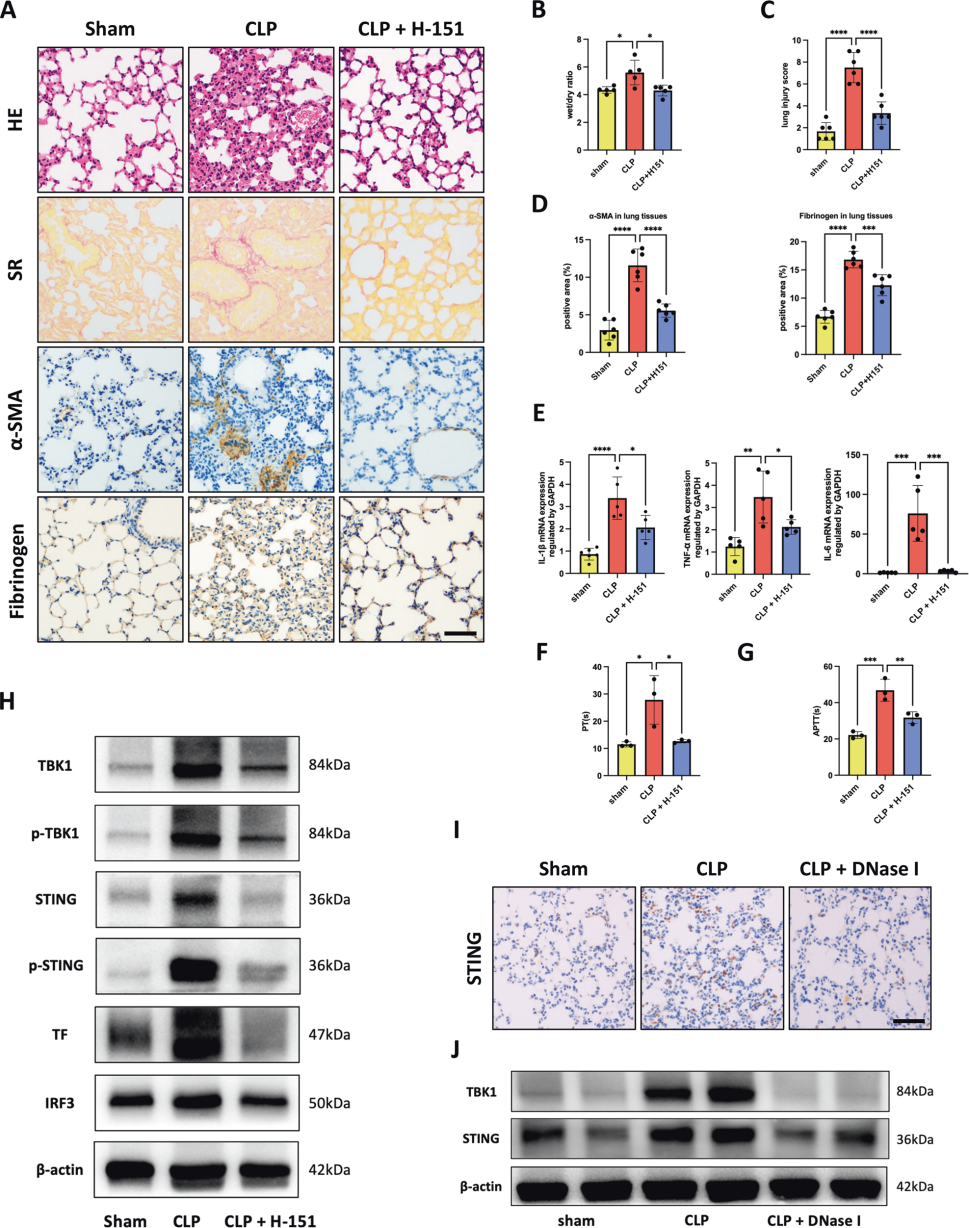
Interruption of the STING pathway alleviated NET-induced TF expression in the CLP mouse model. Interruption of the STING pathway using H-151 protects against lung injury and coagulation cascades induced by CLP. **A** H&E staining, Sirius Red staining, α-SMA and fibrinogen immunohistochemical analysis of the lung tissues. Scale bar: 100 μm. **B** The lung wet/dry ratio (*n* = 6). **C** Lung injury was semi quantified according to H&E staining. The lung injury score was recorded (*n* = 6). **D** The positive area rate of the α-SMA and fibrinogen in lung tissues were calculated by Fiji/ImageJ software (*n* = 6). **E** The mRNA levels of TNF-α, IL-1β, and IL-6 in murine lung tissues (*n* = 5). **F**, **G** PT and APTT were assayed from murine plasma (*n* = 3). **H** Western blot images of STING pathway and TF expression in lung tissues. **I** Representative images of immunohistochemical staining for STING in lung tissues treated with DNase I. Scale bar: 100 μm. **J** Western blot images of the STING pathway in lung tissues treated with DNase I. Each bar represents the mean ± SD. Statistical analysis for three or more groups was carried out using 1-way ANOVA (**B**–**G**). *p < 0.05, **p < 0.01, ***p < 0.001.

To address the role of STING in SI-ALI and coagulation cascades in vivo, mice intraperitoneally received a daily dose of vehicle, H-151, or DNase I starting 24 h before the CLP procedure. Histological examination of the lungs showed significant tissue injury and fibrosis in H-151-treated mice compared to vehicle-treated mice (Fig. 7A, D), accompanied by a reduced lung wet/dry ratio and injury scores (Fig. 7B, C). In addition, STING inhibition reduced the infiltration of inflammatory cells and subsequent cytokine production (Fig. 7E). We observed reduced fibrinogen deposition in lung tissues (Fig. 7A, D) and decreased PT and APTT (Fig. 7F, G). Furthermore, the immunoblotting analysis showed that sepsis-induced TF expression and activation of the STING pathway were attenuated by H-151 treatment (Fig. 7H). However, DNase I administration largely reduced STING activation in lung tissues, suggesting that NETs may interact with the STING pathway (Fig. 7I, J). Taken together, these findings suggested that the STING pathway was responsible for SI-ALI and coagulation dysregulation.

## Discussion

**Fig. 8:**
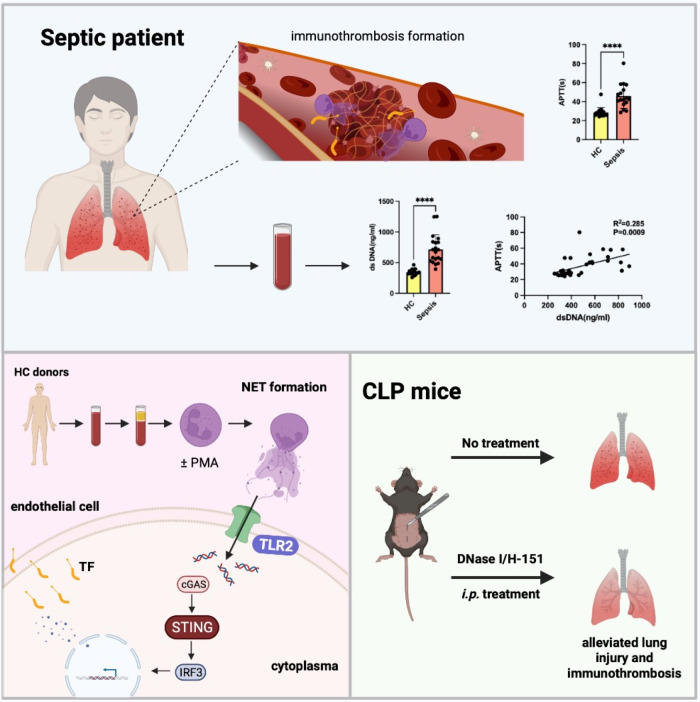
A proposed diagram illustrating how NETs trigger endothelial TF exposure through activation of STING pathway in sepsis.

Immunothrombosis provides an intravascular scaffold for immune cell aggregation, pathogen recognition, and sequestration, but excessive levels contribute to SI-ALI pathogenesis [6]. However, administration of antithrombin [23], recombinant human-activated protein C [24], or TFPI [25] failed to decrease the mortality rate in murine models, indicating that coagulation and inflammation are inextricably correlated in SI-ALI and should not be studied in isolation. In the present study, we found that NETs induce TF expression in endothelial cells through activation of the STING pathway, which suggested intracellular communication between inflammation pathways and coagulation cascades during SI-ALI. Moreover, TLR2 might be required for STING activation in endothelial cells during NET stimulation (Fig. 8).

Numerous studies have revealed that excessive NETs lead to lung injury during sepsis through cell damage and inflammation and coagulation dysregulation, which was triggered by the recruitment of neutrophils and the production of neutrophil-activating factors during pathogen invasion [26]. NET components, including neutrophil elastase, myeloperoxidase, histones, and matrix metalloproteinases, have been proven to damage lung epithelial and endothelial cells through direct cytotoxic activity [27–29]. Moreover, NETs were found to interact with other inflammatory or immune responses, exacerbating septic tissue injury through activation or dysregulation of autophagy [30], ferroptosis [31], apoptosis, and pyroptosis [32]. In this study, we provided several lines of evidence for the activation of STING under NET treatment and its indispensable role in NET-induced endothelial damage and TF expression. Elevated NET formation and STING pathway activation were found in septic patients and SI-ALI model mice, and degradation of NETs greatly mitigated sepsis-induced STING activation, the inflammatory response, and thrombus formation. Furthermore, NET-driven cell damage to endothelial cells, which facilitated the release of TF into the circulation and to the subsequent coagulation cascade, was ameliorated when STING activation was blocked and exacerbated when STING was overexpressed. In vivo, the administration of the STING inhibitor H-151 effectively protected SI-ALI model mice from excessive inflammation and immunothrombosis. STING also served as an important mediator of endothelial cell integrity and the coagulant cascade [33, 34]. Our findings underlined that the interaction between NETs and STING played a role in thrombosis formation. NETs can be recognized by DNA sensors, which, in turn, induces the activation of the STING pathway and production of type I interferons [21]. In tissue plasminogen activator-induced brain hemorrhage after ischemic stroke, activation of the cGAS-STING pathway resulting from NET formation accounted for the deterioration of cerebrovascular integrity [35]. They found that the antihemorrhagic effect of DNase I relied on its ability to inhibit NET-induced cGAS-STING pathway activation, which further extended our knowledge of the interaction between NETs and the STING pathway in specific disease scenarios. In addition, A23187-treated neutrophils releasing NET-like structures containing DNA stimulated macrophages to produce IL-6 and IFN in a cGAS-STING-dependent manner [36], which suggested that the type of NET may also affect their role in the STING pathway. In vivo, Jie Z et al. showed that STING inhibition had little effect on the production and degradation of NETs while DNase I reversed the over-activation of cGAS-STING, connoting STING as downstream of NETs [37]. Interestingly, STING activation induced ROS generation, increased p38 MAPK and ERK1/2 signaling, and subsequently upregulated PAD4 activation, which all participated in NET formation [38]. However, more studies are necessary to elucidate the relationship between NETs and the cGAS-STING pathway in multiple conditions given that cytoplasm mitochondrial DNA or damaged self-DNA could be induced by NETs [37].

KEGG analysis suggested that the TLR signaling pathway was upregulated in NET-treated endothelial cells, and combined qPCR and western blot analysis we screened out that TLR2 was significantly increased under NETs stimulation. Inhibition of NETs reduced TLR2 activation and subsequent cell damage and TF expression, which could also be reduced by TLR2 inhibition and knockdown. Knockout of TLR2 in the CLP mouse model showed ameliorated SI-ALI and coagulation activation, which further strengthened our hypothesis that the interaction between NETs and STING might be partially taken on by TLR2. TLR2 is implicated in the pathogenesis of various diseases through its function within innate immune cells [39, 40]. TLR2 ubiquitously presents within endothelial cells, which are involved in immune cell recruitment and inflammatory and coagulant phenotype transition [41–43]. Neutrophil degranulation triggers oxidative stress, promoting the generation of TLR2 ligands and subsequent activation of TLR2 [44]. Wilson et al. showed that NET- and histone-activated T-cell differentiation was mediated by TLR2 [45], suggesting that TLR2 is important in NET-induced cell communication. Similarly, gastric cells TLR2 responded to NETs and triggered downstream COX-2 release to promote gastric cancer progression [46]. In our study, TLR2 served as a responder of NETs mediating cell injury and coagulant phenotype conversion in endothelial cells. TLR2 recognized and responded to NETs, and activated STING, contributing to TF expression. TLRs were also one of the three major DNA sensors that account for most of our current understanding of DNA­-driven immune responses [16]. For example, TLR9 detected and recognized self-DNA, specifically CpG hypomethylated DNA that enters the cell through the phagolysosomal system [47]. Ds DNA content in NETs might be a potent stimulator of the cGAS-STING pathway, however, it needed more protein interaction experiments to illustrate how TLR2 conveys NETs signal to DNA sensor in the cytoplasm.

TLR2 was predominantly localized on the cell surface, but we observed increased cytoplasmic TLR2 in NET-treated groups. For this phenomenon, we wondered whether the increased cytoplasmic expression of TLR2 was associated with receptor internalization under inflammation. The cellular distribution of TLRs is crucial for its ligand recognition and biological functions, and it could be dynamically altered under peculiar circumstances [48–51]. They exhibit intracellular trafficking, surface expression, internalization, and desensitization. Some of the predominant components in Staphylococcus aureus such as sitC and peptidoglycan could induce intracellular accumulation of TLR2 to sustain subsequent inflammatory responses, which was time and concentration-dependent [52, 53]. Furthermore, RNA-binding protein HuR was found to induce membrane localization of TLR2 through the chaperone CNPY3, and therefore affect TLR2-mediated immune responses and cell functions [54]. Interestingly, in our sequencing data, downregulation of the CNDY3 was found in NET-treated groups, which might be related to increased TLR2 cytosolic localization, but more studies are needed to substantiate it. Functionally, it has been emphasized that TLR2 internalization was required for type I interferon induction [55]. It is possible that increased cytosol TLR2 is necessary for NET-induced STING activation.

Our study has several limitations due to the limited experimental equipment and time. First, NETs can trigger an imbalance between coagulation and anticoagulation and promote a procoagulant endothelial cell phenotype [13]. We mainly focused on TF expression and activity but lacked anti-coagulation indicators. The involvement of intravital microscopy may provide a more holistic and visual formation of immunothrombosis and a possible explanation of the NETs-STING-TF axis in endothelial cells. Second, our experiments were performed in only a single cell line, while the endothelial system was heterogeneous, harboring a specific phenotype and function probably depending on which they are located. Despite this, some of our observations were in accordance with a previous study using human aortic ECs and human saphenous vein ECs to illustrate NETs-induced endothelial dysfunction [56]. Third, although we found that NETs triggered endothelial TF upregulation by STING activation through TLR2, the intrinsic cellular and molecular mechanisms by which STING recognizes NETs and the downstream mechanism by which STING mediates TF expression need further investigation. Last, NET components are complex, and we could not determine which of them serves as the main cause of endothelial and coagulation system homeostasis dysregulation [57]. We took it as a whole and collected NETs from PMA-stimulated neutrophils to treat HUVECs in vitro experiments, while it might be considered limiting and other more physiologically relevant stimuli of NETs were needed [58]. Moreover, it will be meaningful to consider NET interactions in other immune pathways and cell types.

In conclusion, our findings, together with previous studies, emphasized that NETs are key mediators in tissue injury and coagulation, and our study showed that NETs interact with the immune sensor STING through TLR2 to enhance the linkage between inflammation and coagulation by endothelial damage and TF release. Blocking STING-dependent events may maintain a steady state of inflammation and coagulation, demonstrating that it is a potential therapeutic target for SI-ALI.

## Methods

### Ethics statement

The study was approved by the Ethics Committee of Zhongshan Hospital, Fudan University and complied with the principles of the Declaration of Helsinki. Informed consent was obtained from patients or their relatives. All mouse experiments were conducted in accordance with the Regulations for the Administration of Affairs Concerning Experimental Animals and guidelines of the animal review committee of Zhongshan Hospital, Fudan University (Protocol license number: 2020-119).

### Human subjects

The study included patients admitted to the intensive care unit (ICU) from January 2022 to December 2022. Patients were diagnosed with sepsis according to the Third International Consensus Definition for Sepsis [1]^,^ and exclusion criteria included a history of cardiopulmonary arrest, connective tissue disorders, and pregnancy before ICU admission.

### Data collection and blood sample measurements

Patients’ demographic data were recorded following ICU admission. Five milliliters of peripheral venous blood were collected within the first 48 h of diagnosis. Age-matched healthy subjects were enrolled as controls for laboratory studies, and their blood samples were drawn in the morning during fasting conditions. Peripheral blood neutrophil counts, monocyte counts, lymphocyte counts, platelet counts, hemoglobin concentration (g/dl), prothrombin time (in seconds), albumin concentration-activated partial thromboplastin time (in seconds), and fibrinogen and D-dimer concentrations were obtained and recorded.

### Animals

Six- to eight-week-old male TLR2 knockout mice were obtained by crossing male and female heterozygous for C57BL/6JSmoc-Tlr2^em1Smoc^ (Cat. NM-KO-18026, Shanghai Model Organisms, China). C57BL/6J as wild type for KO mice were obtained from Shanghai Laboratory Animal Research Center (Shanghai, China). All animals were maintained under specific pathogen-free conditions in the laboratory animal center of Fudan University.

### Cecal ligation and puncture (CLP) mouse model

After random grouping, the mice were subjected to cecal ligation and puncture procedures to induce sepsis based on previous research [14]. Briefly, a 1–2 cm incision was made in the abdominal wall following intraperitoneal anesthesia with 1% pentobarbital sodium (1 mg/kg). The cecum was carefully exposed and ligated with 5–0 silk sutures 15 mm from the top and punctured through the distal cecum using a 22-gauge needle. Then, we extruded a small quantity of fecal contents, repositioned the cecum into the abdominal cavity, and closed the exposed abdominal wall. Mice allocated to the sham group received the same surgery but without cecal ligation and puncture. All mice were intraperitoneally injected with 0.9% normal saline for rehydration at the end of the surgery and had free access to water and food. If the mice needed treatments, the following reagents were injected intraperitoneally: DNase I (5 mg/kg, 143582, Roche, Mannheim, Germany) and H-151 (750 nM in 200 µl PBS 5% Tween-80, HY-112693, Med Chemical Express, Shanghai, China). The lung tissues were harvested and fixed in 4% paraformaldehyde or immediately stored at −80 °C. Whole blood was centrifuged at 3000 × rpm for 15 min, and serum was collected and stored at −80 °C.

### Histopathological analysis and immunohistochemistry

All mice were sacrificed, and the separated lung tissues were fixed in 4% paraformaldehyde at room temperature for 24 h and then embedded in paraffin. Sections were cut and stained with hematoxylin and eosin (H&E) for histological examination. A semiquantitative scoring system was used to evaluate the severity of lung injury as previously described [30]. Lung injury was scored based on the following criteria: alveolar edema, hemorrhage, leukocyte infiltration, and thickness of the alveolar wall. The scores ranged from 0 to 3 (0 = normal; 1 = mild; 2 = moderate; 3 = severe), and two pathologists blinded to the results graded each indicator and then calculated the total lung injury score.

Lung tissue sections were stained with Sirius red to determine the degree of collagen deposition. For immunohistochemical staining, the slices were dewaxed with xylene and steamed in the citric acid buffer to expose antigen-binding sites. After blocking with 1% bovine serum albumin (BSA, BD Biosciences, New Jersey, USA), primary antibodies against α-SMA, fibrinogen (1:200, Servicebio, Wuhan, China), and STING (1:200, #13647, Cell Signaling Technology, Danvers, MA, USA) were added and incubated overnight at 4 °C. After using HRP-conjugated secondary antibodies, diaminobenzidine staining, and counterstaining with hematoxylin, the expression of a-SMA, fibrinogen, and STING was imaged under light microscopy (Carl Zeiss, Jena, Germany). The positive proportions of the targeted proteins in lung tissues were analyzed by Fiji/ImageJ (NIH, Bethesda, MD) software.

### Lung wet-to-dry ratio

The left lung tissues of mice were harvested. Its wet weight was obtained after absorbing surface water. After drying at 70 °C for 48 h, the dry weight was collected, and the wet/dry weight ratio was calculated by dividing the wet weight by the dry weight.

### Coagulation assay

Blood samples were collected from the posterior eyeball vein of the model mice. The obtained whole blood samples were added into 3.8% sodium citrate anticoagulant tubes according to the 9:1 ratio of blood and anticoagulant, and gently shaken upside-down to avoid clots. Plasma was separated as soon as possible by centrifugation at 3000 × rpm for 15 min, and the upper plasma was collected. Obtained plasma was tested on an automatic coagulation analyzer (RAC-1830, Rayto, Shenzhen, China) using PT assay kit and APTT assay kit (R01002, Rayto).

### Stimulation with PMA and NET release

Human neutrophils were isolated and purified according to the kit’s instructions (TBD Sciences, Tianjin, China). The blood samples (5 ml each) were layered over neutrophil separation medium (5 ml each) and centrifuged at 3000 rpm for 30 min. We carefully collected the lower leukocyte layer containing neutrophils and eliminated erythrocytes with a hypotonic lysis buffer. The obtained cells were resuspended in DMEM (Gibco, USA) supplemented with 10% FBS and seeded in 6-well plates at a density of 1 × 10^6^ cells per ml. Neutrophils were stimulated with 100 nM PMA (MKbio, Shanghai, China) for 4 h at 37 °C. Then, we removed the supernatant, washed the cells twice with cold PBS, and added 2 ml of cell culture medium to agitate the NETs adhered at the bottom. The NETs were collected in 15-ml tubes and centrifuged at 300 × *g* for 5 min, and we collected the supernatant for immediate use or snap frozen in liquid nitrogen.

### Quantification of dsDNA

DNA in the plasma of human and mice was quantified according to the manufacturer’s instructions using Quant-iT^TM^ PicoGreen ® dsDNA Reagent and Kits (Invitrogen, MA, USA). The fluorescence intensity reflects the amount of DNA, which was measured at excitation and emission wavelengths of 480 nm and 520 nm, respectively, in a microplate reader (Thermo Fisher Scientific, MA, USA).

### Measuring MPO-DNA complexes

The MPO-DNA complexes concentration in the serum of human and mice was measured using a capture ELISA kit. Briefly, For the capture of antibody, 5 μg/ml anti-MPO monoclonal antibody (Proteintech, 22225-1-AP, Shanghai, China) was coated onto 96-well microtiter plates overnight at 4 °C. After blocking in 1% BSA, serum was added to the wells with peroxidase-labeled anti-DNA monoclonal antibody (Cell Death Detection ELISA kit, Roche). The plate was incubated for two hours at room temperature and washed with PBS three times. Next, the peroxidase substrate (ABTS) was added. After 40 mins of incubation at 37 °C in the dark, the optical density (OD) was measured at 405 nm using a microplate reader (Thermo Fisher Scientific).

### Cell culture and treatments

HUVECs were obtained from American Type Culture Collection (ATCC; Manassas, USA) and cultured in DMEM (Gibco) with 5 mM glucose supplemented with 10% fetal bovine serum (Gibco) and penicillin/streptomycin (Gibco, 100 g/ml) at 37 °C in a humidified 5% carbon dioxide incubator. The STING inhibitor H-151 (HY-112693, 10 μM, 2 h) and TLR2 inhibitor C29 (HY-100461, 20 μM, 2 h) were purchased from Med Chemical Express (Shanghai, China). HUVECs were transfected with Lentivirus-NC or Lentivirus-STING (MOI = 50; Shanghai GeneChem, China) to overexpress STING for 72 h and the sequence was listed in supplementary table 3. Then the culture medium was replaced and cultured after 3 days for the subsequent experiment. The transfection efficacy was assessed by qPCR and western blot. Lentivirus-NC-RNAi or Lentivirus-TLR2-RNAi were purchased from Shanghai GeneChem (MOI = 30), and the sequence of TLR2-RNAi was 5’- CAGGATCACCTACATTAGCAA-3’. HUVECs were transfected according to the manufacturer’s instructions for further studies.

### Cell viability

A Cell-Counting Kit 8 (Dojindo Corp., Kumamoto, Japan) was used to test relative cell viability at selected times according to the manufacturer’s instructions. Briefly, Cells were seeded in 96-well plates (5 × 10^3^ cells/well) with the corresponding treatment, and the culture supernatant was converted to 10% CCK-8 fresh medium at the pointed time. The absorbance at 450 nm was determined in a multimode microplate reader (Thermo Fisher Scientific).

### Western blotting

Whole cells were washed with ice-cold phosphate-buffered saline and lysed in RIPA buffer (Solarbio, Beijing, China) containing proteinase inhibitor cocktails. Protein samples were separated by 7.5–12.5% sodium dodecyl sulfate-polyacrylamide gel electrophoresis (SDS‒PAGE) and transferred to polyvinylidene fluoride membranes (Millipore, Billerica, USA). The membranes were blocked with Tris-buffered saline Tween 20 (TBST, Sangon Biotech, Shanghai, China) containing 5% skim milk (BD Biosciences) and incubated with primary antibodies against MPO (1:1000, ab208670), TF (1:1000, ab189483, ab228968), STING (1:1000, ab239074), TBK1 (1:1000, ab40676), TLR1 (1:1000, ab68158), TLR2 (1:1000, ab209216), TLR3 (1:1000, ab307442), TLR5 (1:1000, ab168382), TLR7 (1:1000, ab124928), TLR8(1:1000, ab228962, Abcam, Cambridge, MA, USA), TLR4 (1:1000, AF7017), TLR9 (1:1000, DF2970, Affinity, Jiangsu, China), TLR6 (1:1000, 22410-1-AP, Proteintech), TLR10 (1:150, MAB6619-SP, R&D Systems Inc., Minneapolis, MN, USA), p-STING (1:1000, #19781), p-TBK1 (1:1000, #5483), IRF3 (1:1000, #4302), p-IRF3 (1:1000, #37829), cGAS (1:1000, #79978, Cell Signaling Technology), TLR2 (1:1000, MA5-32787, Invitrogen), β-actin (1:5000, 100166-MM10, Sino Biological, Beijing, China), GAPDH (1:30000, AC033, ABclonal, Wuhan, China) overnight at 4 °C. After being washed with TBST for three times, the membranes were incubated with HRP-linked anti-mouse IgG secondary antibody (1:3000; A0216, Beyotime, Shanghai, China) or HRP-linked anti-rabbit IgG secondary antibody (1:1000; 7074, Cell Signaling Technology) for 1 h at room temperature. The membranes were washed and detected with an ECL chemiluminescence kit (Millipore), and the bands of the target proteins were visualized by the LAS-3000 detection system and were quantitively analyzed by Fiji based on β-actin and control groups respectively.

### Immunofluorescence (IF) staining

Cells were seeded in 24-well plates (5 × 10^4^ cells/well) with the corresponding treatment. After fixation with 4% paraformaldehyde, the cells were permeabilized with 0.1% Triton X-100 for 5 min and then blocked for 30 min at room temperature. The cells were incubated with antibodies against F-actin (1:100, C2201S, Beyotime), TF (1:100, ab228968, Abcam), STING (1:100, ab239074, Abcam), TLR2 (1:150, MA5-32787, Invitrogen) overnight at 4 °C, and washed before incubated with fluorescent Alexa Fluor® 488-conjugated goat anti-mouse IgG (1:200, ab150113, Abcam) or Alexa Fluor® 594-conjugated goat anti-rabbit IgG (1:200, ab150080, Abcam). 4,6-diamidino-2-phenylindole (DAPI) was used for nuclear staining. To visualize NETs in lung tissue samples of the CLP mouse model, paraffin-embedded tissue sections were deparaffinized, rehydrated, and processed with heat induction for antigen retrieval. They were incubated with primary antibodies against citH3 (1:100, ab5103, Abcam), and MPO (1:100, ab90810, Abcam) after blocking, and DAPI was used to stain the nuclei. Images were taken under an Olympus microscope (Tokyo, Japan). The relative fluorescence intensity of targeted proteins was quantified using Fiji/ ImageJ software.

### RNA extraction and quantitative real-time PCR analysis

Total RNA was extracted from tissues or cells using TRIzol reagent (Invitrogen) and purified RNA (500 ng) was reverse-transcribed into cDNA using the PrimeScript RT reagent kit (Takara, Shinga, Japan) according to the manufacturer’s instructions. qPCR was then performed on 1 μg of cDNA using a TB Green PCR kit (Takara). Primers were synthesized by TSINGKE Biological Technology (Nanjing, China), and the sequences of the primers are listed in Supplementary Table 2. Fold changes in the expression levels were compared with those of GAPDH.

### LDH-Glo^TM^ cytotoxicity assay

Cells were cultured in 96-well plates. After NET stimulation, the supernatants of cells were collected for analysis of LDH levels using an LDH Cytotoxicity Assay Kit (Promega, Wisconsin, USA) according to the manufacturer’s protocol. Briefly, 50 μl of supernatant samples were transferred to a 96-well plate and mixed with 50 μl of the LDH reagent. After incubating at room temperature for 30 min, the LDH activity was measured in a multimode microplate reader (PerkinElmer, Waltham, MA, USA).

### RNA-seq analysis

Total RNA was extracted using TRIzol reagent (Invitrogen), and the purity, concentration, and integrity of the RNA were evaluated for further library construction. Poly(A) RNA was subsequently purified and used to generate cDNA libraries. All samples were sequenced using an MGISEQ-T7 instrument (BGI), and 150 bp paired-end reads were generated. Next, the raw reads were filtered by removing adapter and poly-N sequences and inferior-quality reads to obtain clean reads, which were separately aligned to the reference genome in orientation mode using HISAT2 software. Then, FeatureCounts (http://subread.sourceforge.net) was applied to quantify the read numbers mapped to each gene. Differential expression analysis was performed using DESeq2, and genes with *P* < 0.05 were defined as differentially expressed genes (DEGs). The KEGG plot was generated using BioLadder online tools (https://www.bioladder.cn).

### Statistical analysis

All experimental data were expressed as the mean ± standard deviation (SD). Student’s *t* test was applied for comparisons between two groups and one-way ANOVA followed by Tukey’s post hoc test for three or more groups. Statistical analyses were carried out using SPSS 23.0 and GraphPad Prism 9.0 software. Differences where *P* < 0.05 were considered statistically significant. All experiments were repeated at least three times independently.

### Supplementary information

supplementary figure 1

Supplementary Figure Legends

Supplementary Table 1

Supplementary Table 2

Supplementary Table 3

Original Data File

## Data Availability

All relevant data are enclosed in this published article and its supplementary files.
