# Supplementary figures and images for: Neutrophil extracellular traps contribute to immunothrombosis formation via the STING pathway in sepsis-associated lung injury
Source: Cell Death Discov. 2023 Aug 25;9:315. doi: 10.1038/s41420-023-01614-8 (PMC10457383; doi:10.1038/s41420-023-01614-8)

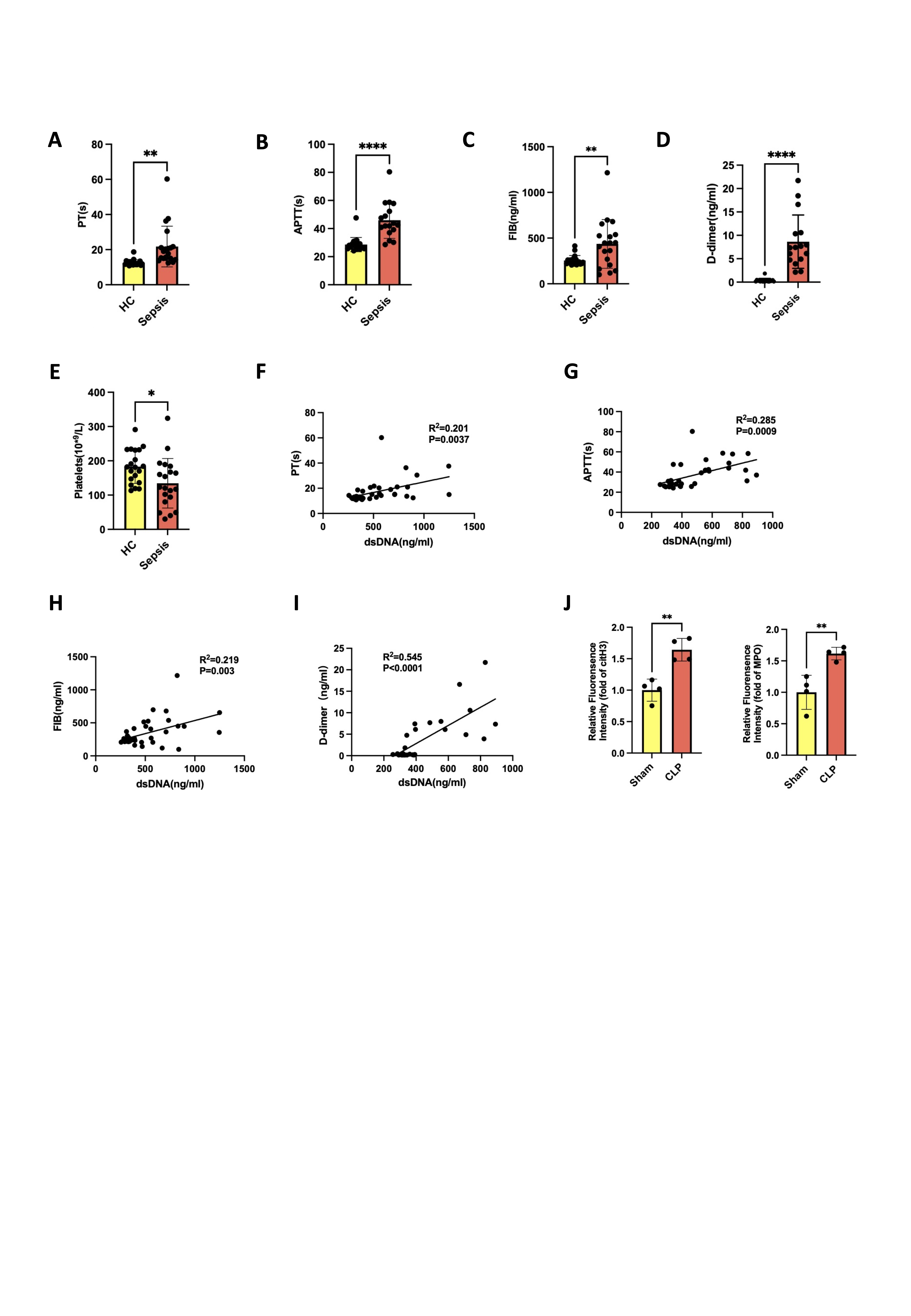

Supplement: Supplementary file 1 — supplementary figure 1 [file 41420_2023_1614_MOESM1_ESM.png]
