# Supplementary material for: Neutrophil extracellular traps contribute to immunothrombosis formation via the STING pathway in sepsis-associated lung injury
Source: Cell Death Discov. 2023 Aug 25;9:315. doi: 10.1038/s41420-023-01614-8 (PMC10457383; doi:10.1038/s41420-023-01614-8)
Supplement: Supplementary file 2 — Supplementary Figure Legends [file 41420_2023_1614_MOESM2_ESM.docx]

**Figure Legends**

**Supplementary Fig. 1 related to Fig 1.** **NET formation correlated with SI-ALI and coagulation cascades A-E** Plasma levels of procoagulant markers, including prothrombin time (PT), activated partial thromboplastin time (APTT), fibrinogen (FIB), D-dimer, and platelet numbers, were assayed from septic patients (n=20) and HCs (n=20). **F-I** Correlation curves between dsDNA and PT, APTT, FIB, and D-dimer. **J** The expression of citH3 and MPO was assessed by fluorescence intensity (n=4). Each bar represents the mean ± SD. The comparison between the two groups was performed using unpaired t-tests (**A**-**E**, and **J**). **p* < 0.05, ***p* < 0.01, ****p* < 0.001.
