# Supplementary material for: Neutrophil extracellular traps contribute to immunothrombosis formation via the STING pathway in sepsis-associated lung injury
Source: Cell Death Discov. 2023 Aug 25;9:315. doi: 10.1038/s41420-023-01614-8 (PMC10457383; doi:10.1038/s41420-023-01614-8)
Supplement: Supplementary file 3 — Supplementary Table 1 [file 41420_2023_1614_MOESM3_ESM.docx]

**Supplementary Table 1. Baseline characteristics of healthy controls (HC) and Sepsis patients.**

|  | Healthy Control  (n = 20) | Sepsis  (n=20) | P value |
| --- | --- | --- | --- |
| Gender (n, %) |  |  | 0.67 |
| Male | 13 (65.0%) | 15 (75.0%) |  |
| Female | 7 (35.0%) | 5 (25.0%) |  |
| Ages (years) | 53.5±8.3 | 59.6±12.3 | 0.09 |
| BMI (kg/m^2^) | 23.6±3.7 | 22.4±3.4 | 0.297 |
| Neutrophils (10^9^/L) | 3.6±0.7 | 7.6±2.9 | <0.05 |
| Monocytes (10^9^/L) | 0.52±0.23 | 0.63±0.51 | 0.39 |
| Lymphocytes (10^9^/L) | 1.78±0.48 | 0.71±0.44 | <0.05 |
| Platelets (10^9^/L) | 183.2±49.25 | 134.4±72.34 | <0.05 |
| Hemoglobin (g/L) | 128.3±19.72 | 83.65±20.60 | <0.05 |
| Albumin (g/L) | 42.8±3.92 | 32.75±4.05 | <0.05 |
| PT (s) | 12.53±1.719 | 21.77±11.58 | <0.05 |
| APTT (s) | 18.61±5.062 | 45.97±12.90 | <0.05 |
| Fibrinogen (g/L) | 255.1±54.83 | 436.5±266.5 | <0.05 |
| D-dimer (ng/ml) | 0.332±0.391 | 8.64±5.70 | <0.05 |
| PaO_2_/FiO_2_ (mmHg) | 527.0±88.03 | 320±137.8 | <0.05 |

Data are expressed as n (%), mean ± SD or median (interquartile range [IQR]). BMI: body mass index.
