# Supplementary material for: Neutrophil extracellular traps contribute to immunothrombosis formation via the STING pathway in sepsis-associated lung injury
Source: Cell Death Discov. 2023 Aug 25;9:315. doi: 10.1038/s41420-023-01614-8 (PMC10457383; doi:10.1038/s41420-023-01614-8)
Supplement: Supplementary file 4 — Supplementary Table 2 [file 41420_2023_1614_MOESM4_ESM.docx]

**Supplementary Table 2. Primer sequences for RT-qPCR.**

| Gene | Forward | Reverse |
| --- | --- | --- |
| Human-TLR1 | CCACGTTCCTAAAGACCTATCCC | CCAAGTGCTTGAGGTTCACAG |
| Human-TLR2 | ATCCTCCAATCAGGCTTCTCT | GGACAGGTCAAGGCTTTTTACA |
| Human-TLR3 | TTGCCTTGTATCTACTTTTGGGG | TCAACACTGTTATGTTTGTGGGT |
| Human-TLR4 | AGACCTGTCCCTGAACCCTAT | CGATGGACTTCTAAACCAGCCA |
| Human-TLR5 | GCCGGTCCTGTGTTTGGAAT | GGTGAGGTTGCAGAAACGATAAA |
| Human-TLR6 | TTCTCCGACGGAAATGAATTTGC | CAGCGGTAGGTCTTTTGGAAC |
| Human-TLR7 | TCCTTGGGGCTAGATGGTTTC | TCCACGATCACATGGTTCTTTG |
| Human-TLR8 | ATGTTCCTTCAGTCGTCAATGC | TTGCTGCACTCTGCAATAACT |
| Human-TLR9 | CTGCCTTCCTACCCTGTGAG | GGATGCGGTTGGAGGACAA |
| Human-TLR10 | AGGTTTGAGTGGGGCAAAAAT | CCATCACGCAAAAGAACCCAG |
| Human-TF | GGCGCTTCAGGCACTACAA | TTGATTGACGGGTTTGGGTTC |
| Human-STING | CCAGAGCACACTCTCCGGTA | CGCATTTGGGAGGGAGTAGTA |
| Human-TBK1 | TGCACCCTGATATGTATGAGAGA | AAATGGCAGTGATCCAGTAGC |
| Human-IL-6 | CAGCCCTGAGAAAGGAGACAT | GGTTCAGGTTGTTTTCTGCCA |
| Human-GAPDH | GGAGCGAGATCCCTCCAAAAT | GGCTGTTGTCATACTTCTCATGG |
| Mouse-TF | CTGGAAAAACAAGTGCTTCTCG | ACAGAGAGGACCTTTGCTTCA |
| Mouse-IL-1β | GAAATGCCACCTTTTGACAGTG | TGGATGCTCTCATCAGGACAG |
| Mouse-IL-6 | CTGCAAGAGACTTCCATCCAG | AGTGGTATAGACAGGTCTGTTGG |
| Mouse-TNF-α | CAGGCGGTGCCTATGTCTC | CGATCACCCCGAAGTTCAGTAG |
| Mouse-TLR2 | TCTAAAGTCGATCCGCGACAT | CTACGGGCAGTGGTGAAAACT |
| Mouse-GAPDH | TGACCTCAACTACATGGTCTACA | CTTCCCATTCTCGGCCTTG |
