# Supplementary material for: Neutrophil extracellular traps contribute to immunothrombosis formation via the STING pathway in sepsis-associated lung injury
Source: Cell Death Discov. 2023 Aug 25;9:315. doi: 10.1038/s41420-023-01614-8 (PMC10457383; doi:10.1038/s41420-023-01614-8)
Supplement: Supplementary file 5 — Supplementary Table 3 [file 41420_2023_1614_MOESM5_ESM.docx]

**The sequences of lentivirus-STING:**

Insertion sequences are in blue font, vector sequences are in black font, and restriction sites are underlined.

GAACTATGCGCTCGGGGTTGGCGAGTGTGTTTTGTGAAGTTTTTTAGGCACCTTTTGAAATGTAATCATTTGGGTCAATATGTAATTTTCAGTGTTAGACTAGTAAATTGTCCGCTAAATTCTGGCCGTTTTTGGCTTTTTTGTTAGACGAAGCTTGGGCTGCAGGTCGACTCTAGAGGATCCCCGGGTACCGGTCGCCACCATGCCCCACTCCAGCCTGCATCCATCCATCCCGTGTCCCAGGGGTCACGGGGCCCAGAAGGCAGCCTTGGTTCTGCTGAGTGCCTGCCTGGTGACCCTTTGGGGGCTAGGAGAGCCACCAGAGCACACTCTCCGGTACCTGGTGCTCCACCTAGCCTCCCTGCAGCTGGGACTGCTGTTAAACGGGGTCTGCAGCCTGGCTGAGGAGCTGCGCCACATCCACTCCAGGTACCGGGGCAGCTACTGGAGGACTGTGCGGGCCTGCCTGGGCTGCCCCCTCCGCCGTGGGGCCCTGTTGCTGCTGTCCATCTATTTCTACTACTCCCTCCCAAATGCGGTCGGCCCGCCCTTCACTTGGATGCTTGCCCTCCTGGGCCTCTCGCAGGCACTGAACATCCTCCTGGGCCTCAAGGGCCTGGCCCCAGCTGAGATCTCTGCAGTGTGTGAAAAAGGGAATTTCAACGTGGCCCATGGGCTGGCATGGTCATATTACATCGGATATCTGCGGCTGATCCTGCCAGAGCTCCAGGCCCGGATTCGAACTTACAATCAGCATTACAACAACCTGCTACGGGGTGCAGTGAGCCAGCGGCTGTATATTCTCCTCCCATTGGACTGTGGGGTGCCTGATAACCTGAGTATGGCTGACCCCAACATTCGCTTCCTGGATAAACTGCCCCAGCAGACCGGTGACCATGCTGGCATCAAGGATCGGGTTTACAGCAACAGCATCTATGAGCTTCTGGAGAACGGGCAGCGGGCGGGCACCTGTGTCCTGGAGTACGCCACCCCCTTGCAGACTTTGTTTGCCATGTCACAATACAGTCAAGCTGGCTTTAGCCGGGAGGATAGGCTTGAGCAGGCCAAACTCTTCTGCCGGACACTTGAGGACATCCTGGCAGATGCCCCTGAGTCTCAGAACAACTGCCGCCTCATTGCCTACCAGGAACCTGCAGATGACAGCAGCTTCTCGCTGTCCCAGGAGGTTCTCCGGCACCTGCGGCAGGAGGAAAAGGAAGAGGTTACTGTGGGCAGCTTGAAGACCTCAGCGGTGCCCAGTACCTCCACGATGTCCCAAGAGCCTGAGCTCCTCATCAGTGGAATGGAAAAGCCCCTCCCTCTCCGCACGGATTTCTCTTGACTAGCCTGTGGAATGTGTGTCAGTTAGGGTGTGGAAAGTCCCCAGGCTCCCCAGCAGGCAGAAGTATGCAAAGCATGCATCTCAATTAGTCAGCAACCAGGTGTGGAAAGTCCCCAGGCTCCCCAGCAGGCAGAAGTATGCAAAGCATGCATCTCAATTAGTCAGCAACCATAGTCCCGCCCCTAACTCCGCCCATCCCGCCCCTAACTCCGCCCAGTTCCGC
