# Supplementary material for: Neutrophil extracellular traps contribute to immunothrombosis formation via the STING pathway in sepsis-associated lung injury
Source: Cell Death Discov. 2023 Aug 25;9:315. doi: 10.1038/s41420-023-01614-8 (PMC10457383; doi:10.1038/s41420-023-01614-8)

Figure 1. M

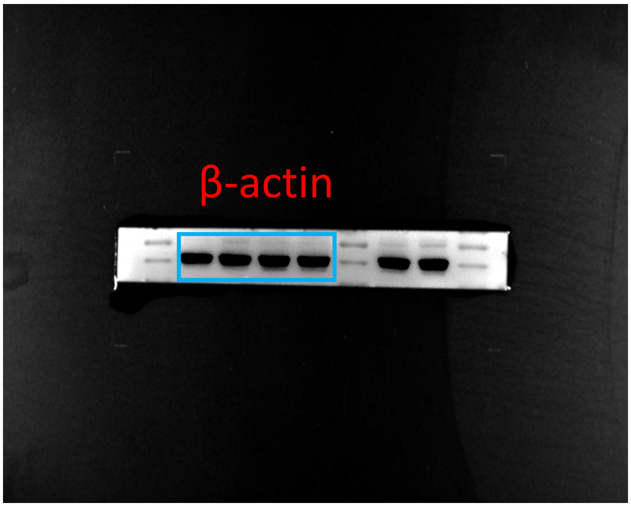

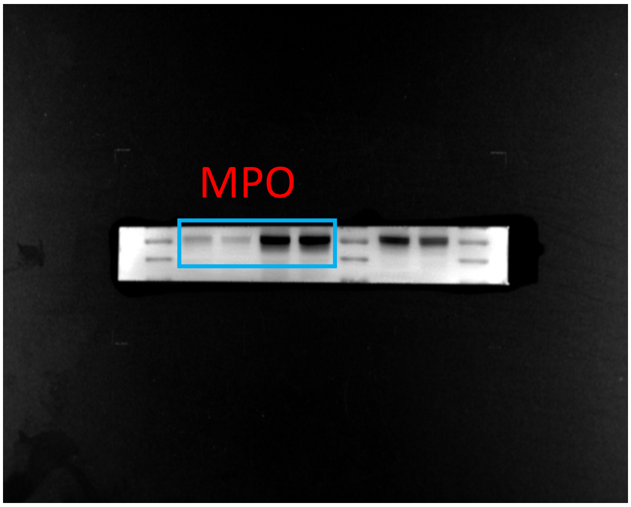

Figure 1. N

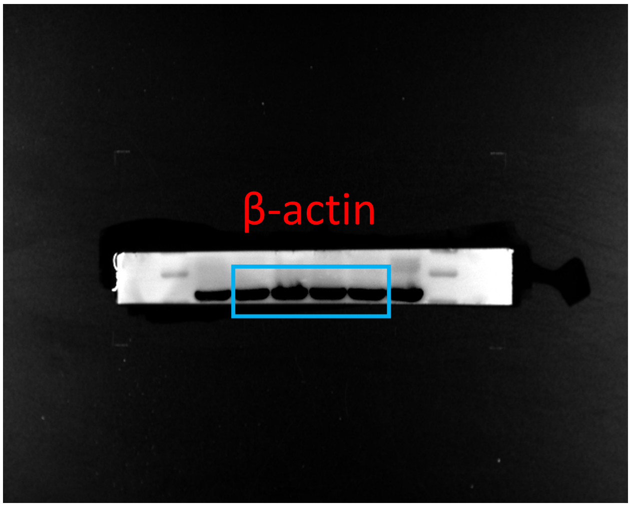

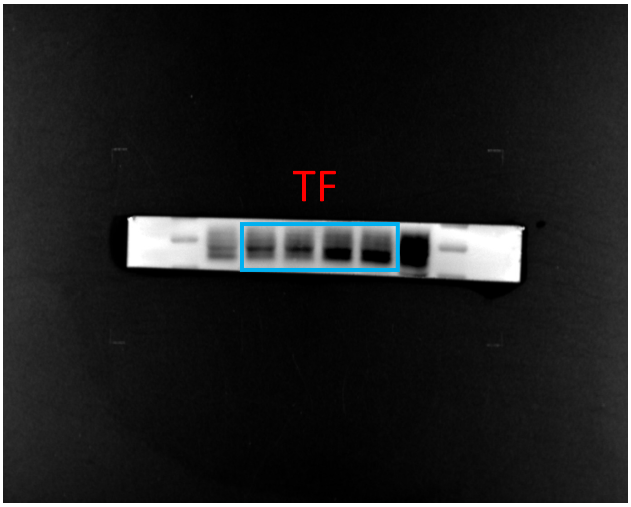

Figure 2. F

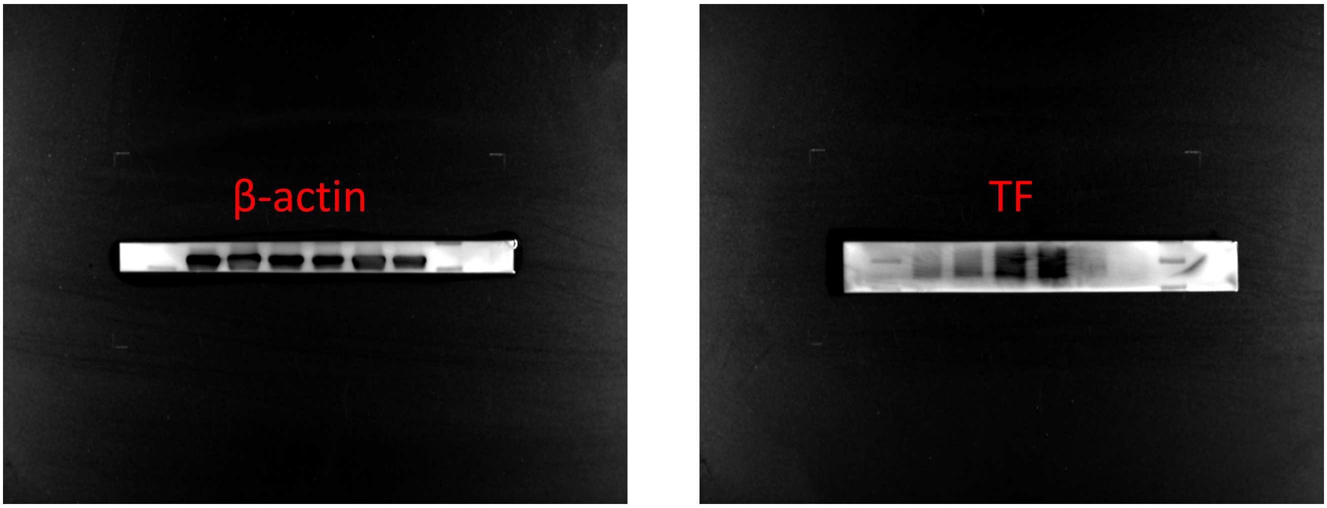

Figure 3. E

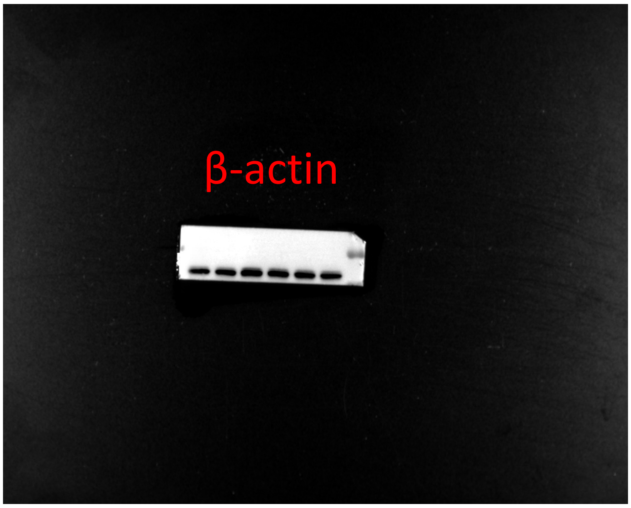

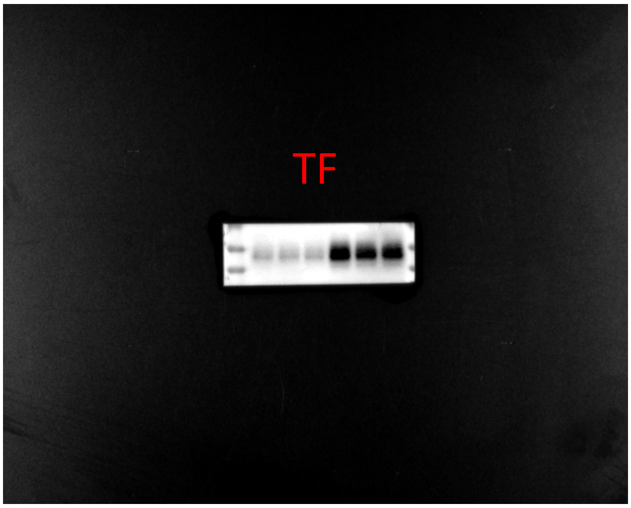

Figure 3. H

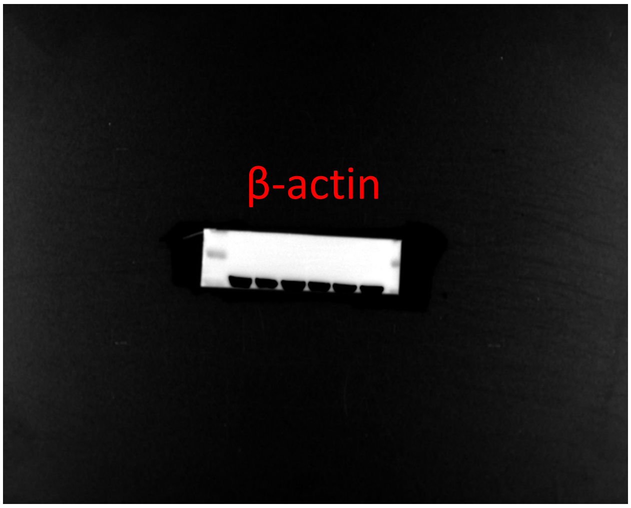

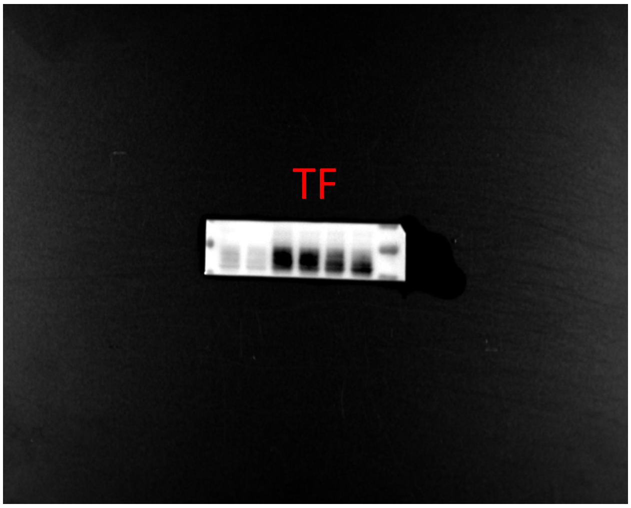

Figure 4. A

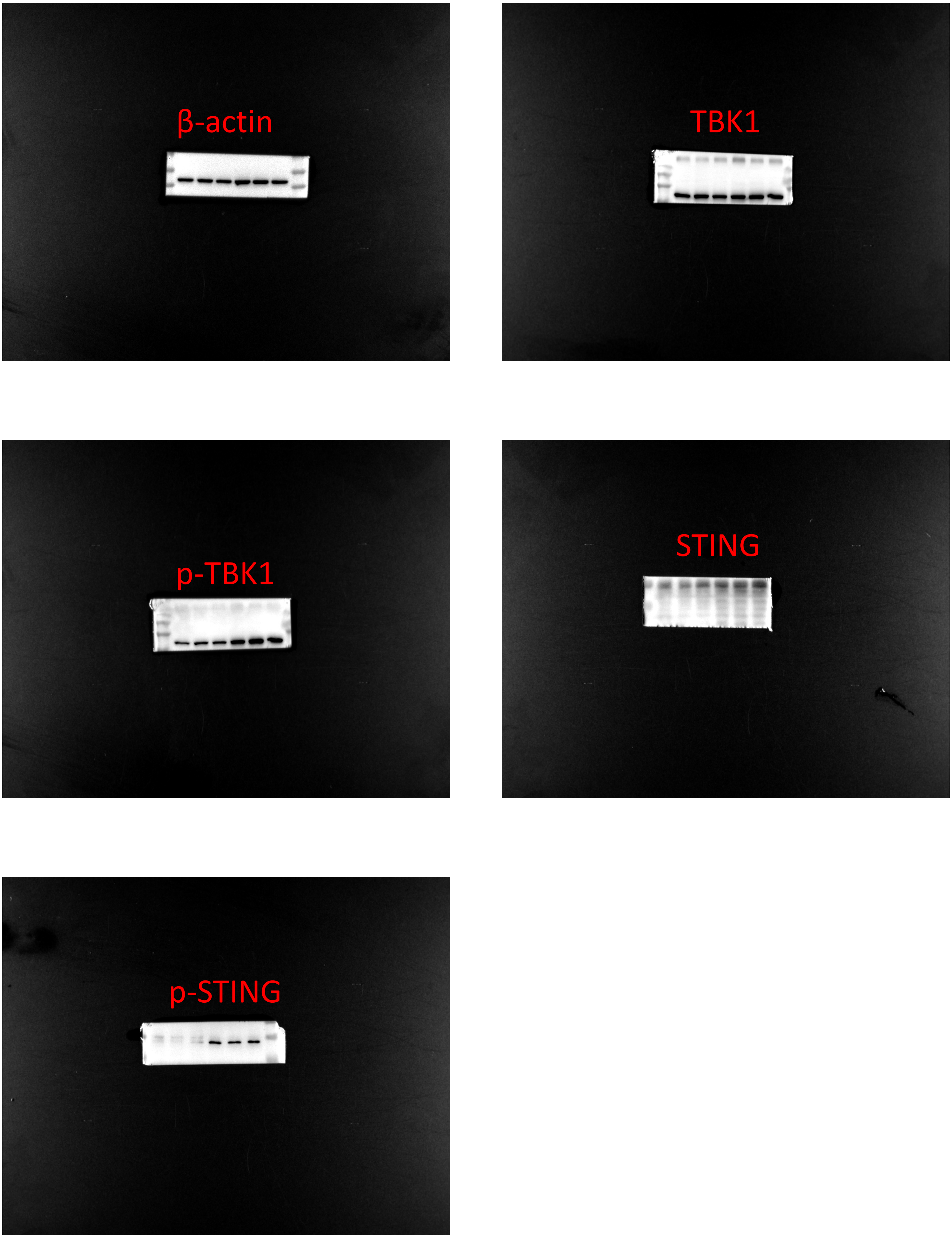

Figure 4. G

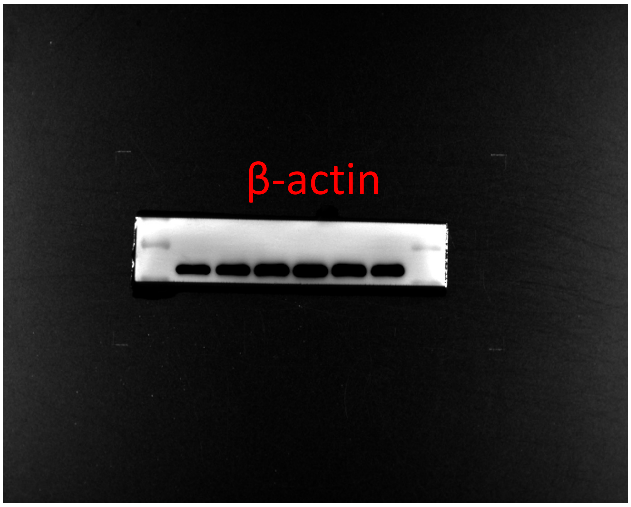

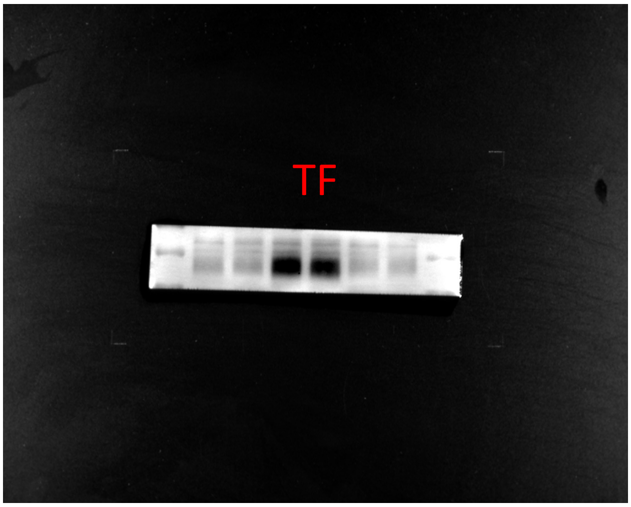

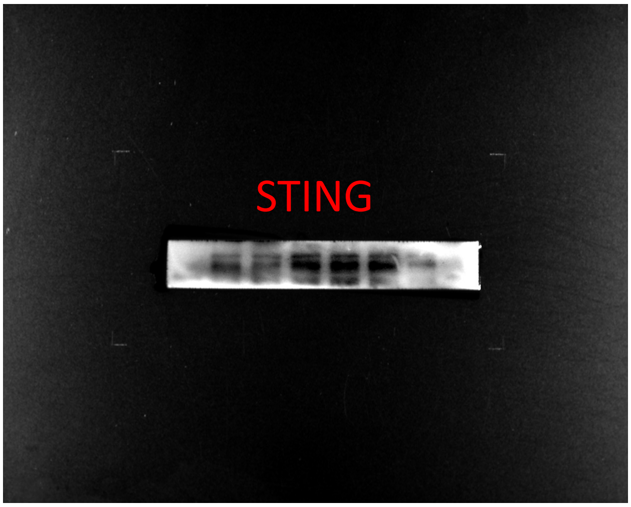

Figure 4. G

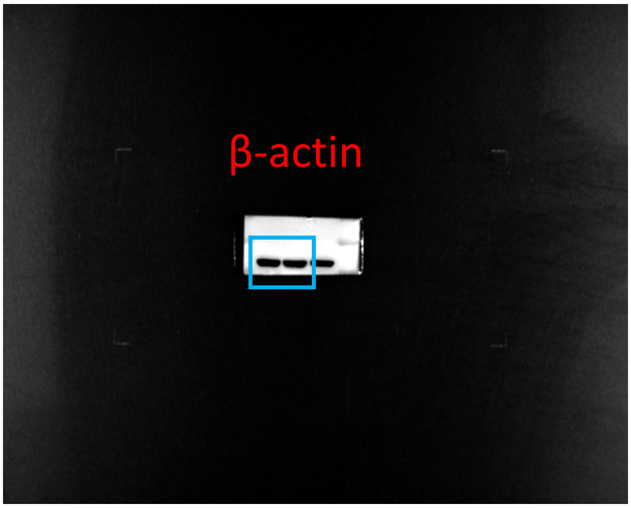

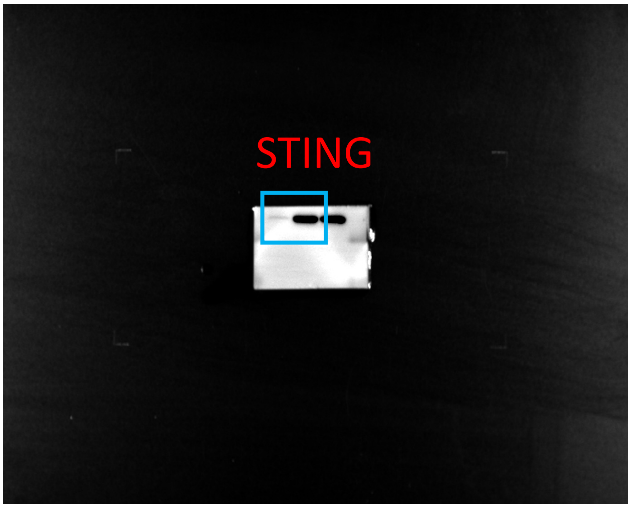

Figure 4. J

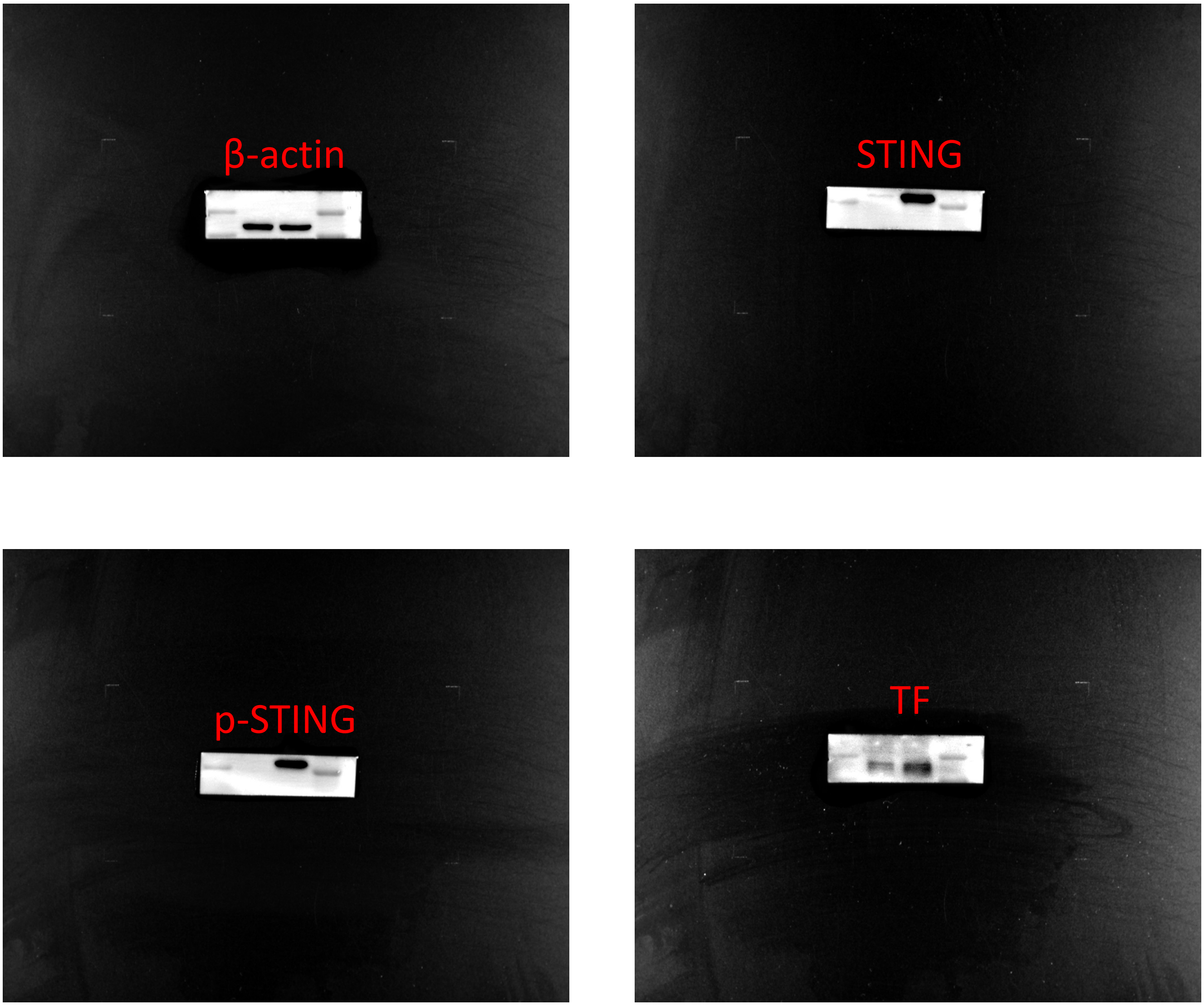

Figure 5. C

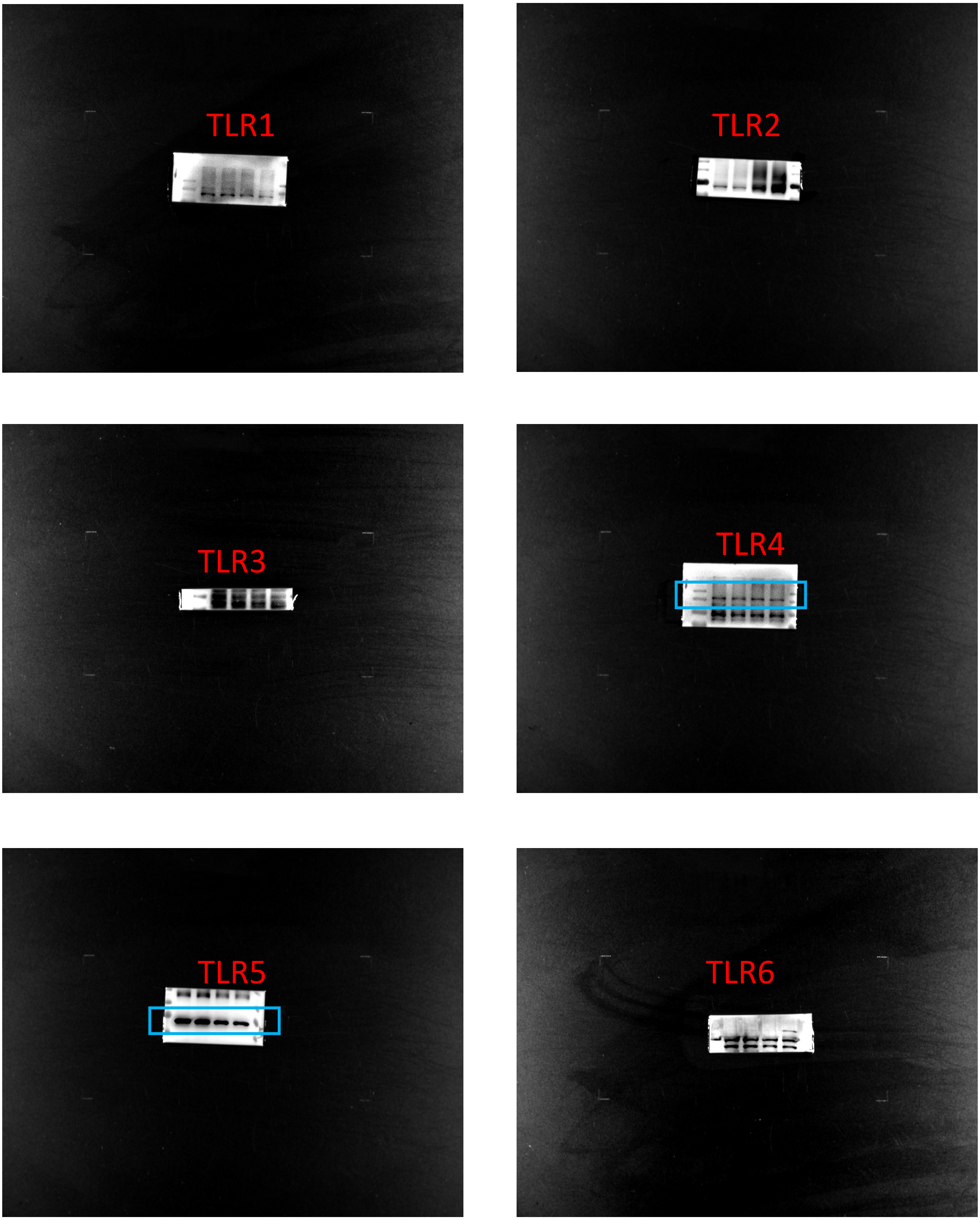

Figure 5. C-continued

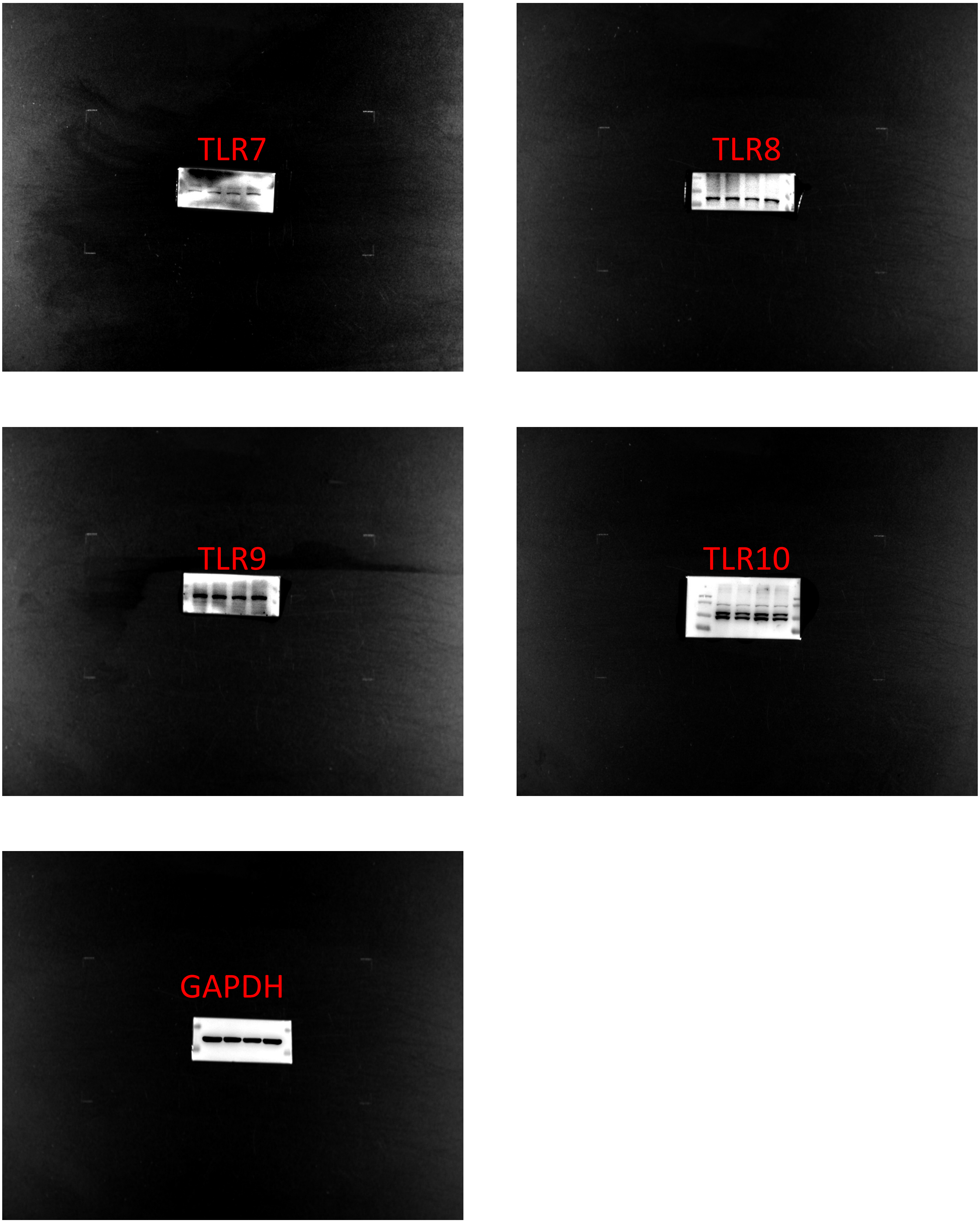

Figure 5. G

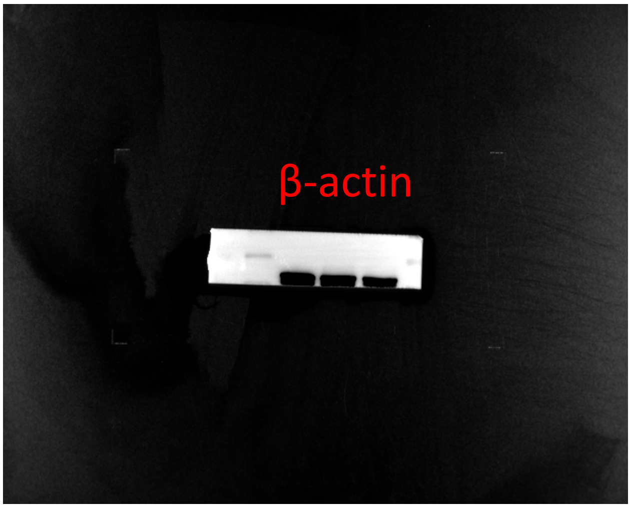

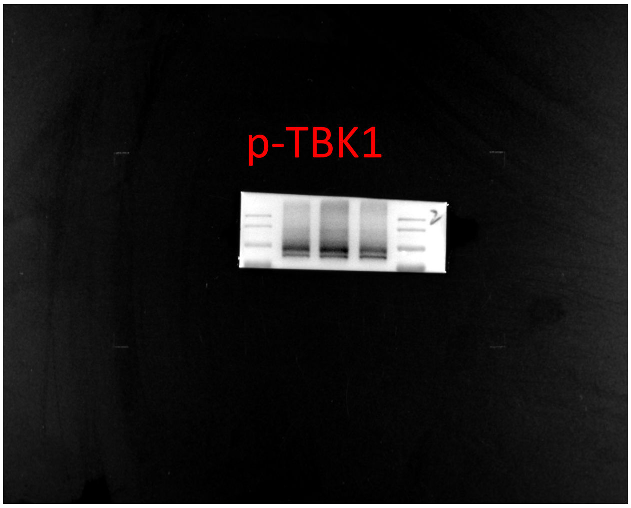

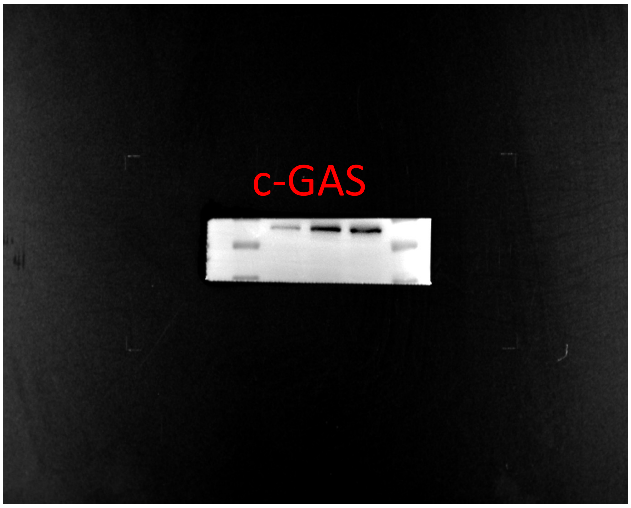

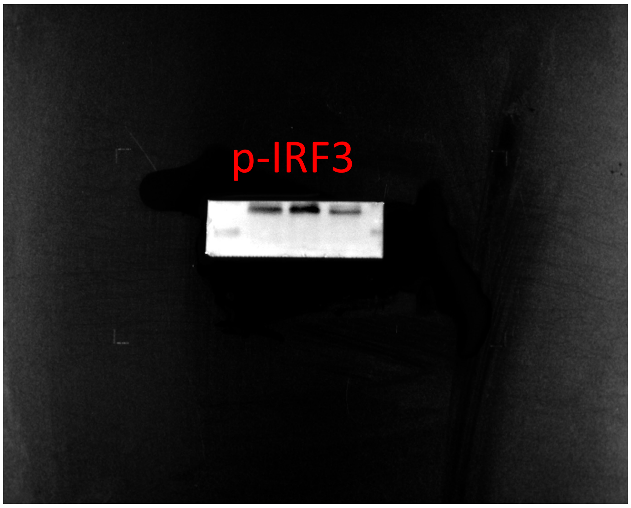

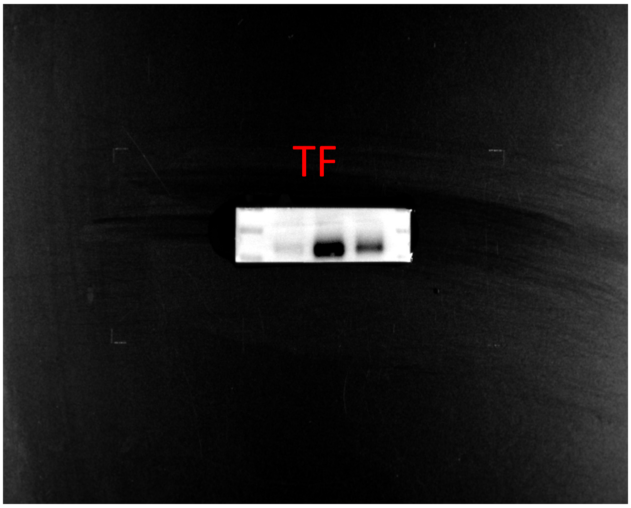

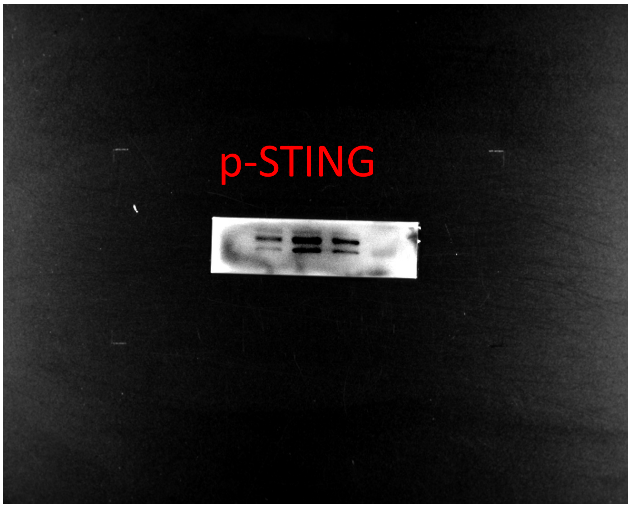

Figure 5. G-continued

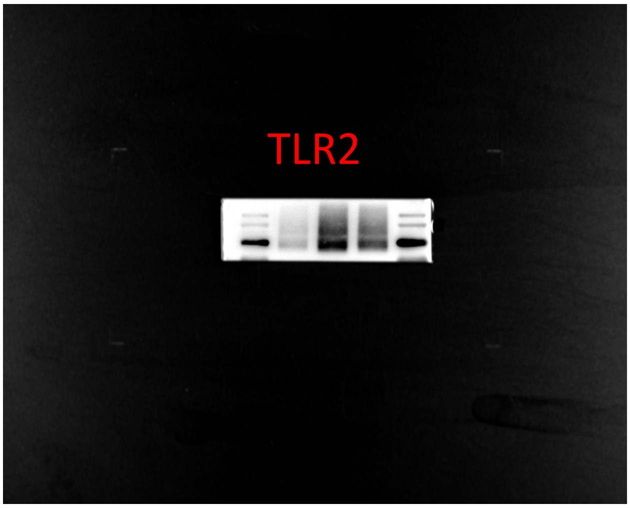

Figure 5. I

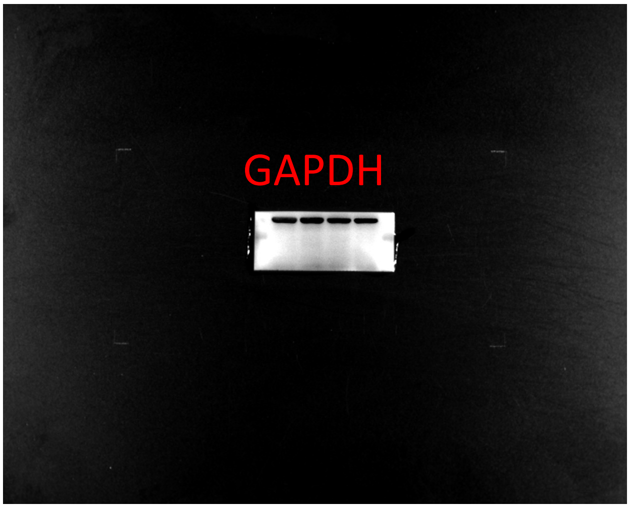

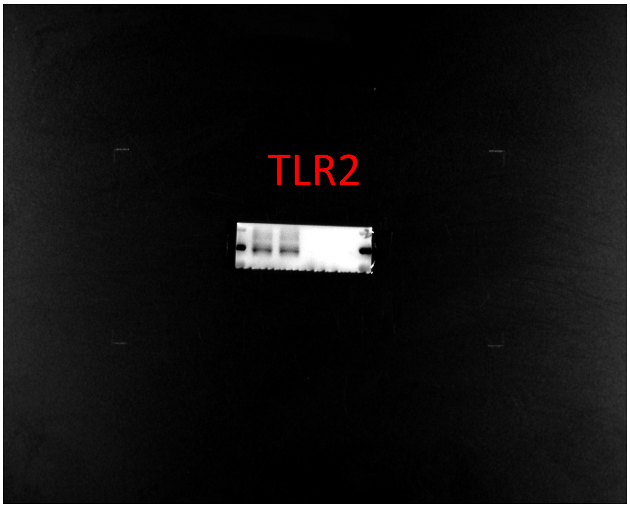

Figure 5. J

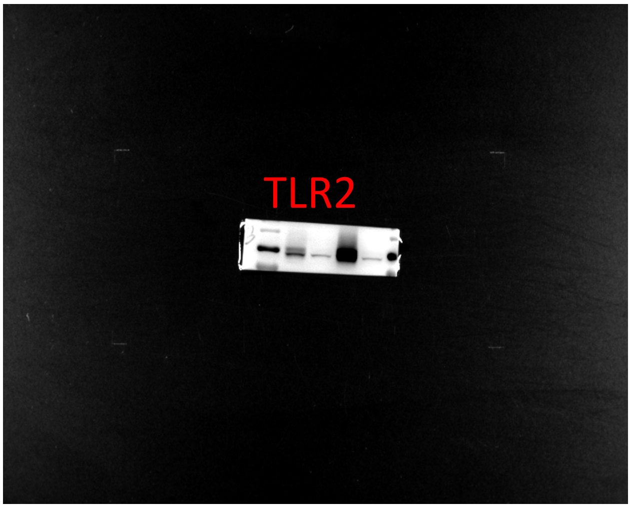

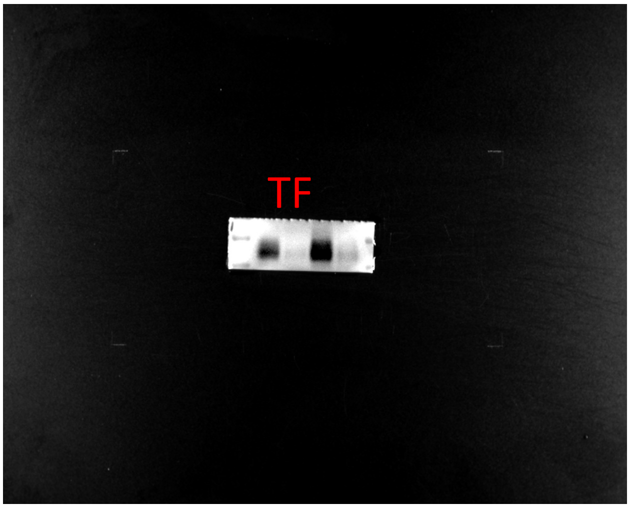

Figure 5. J-continued

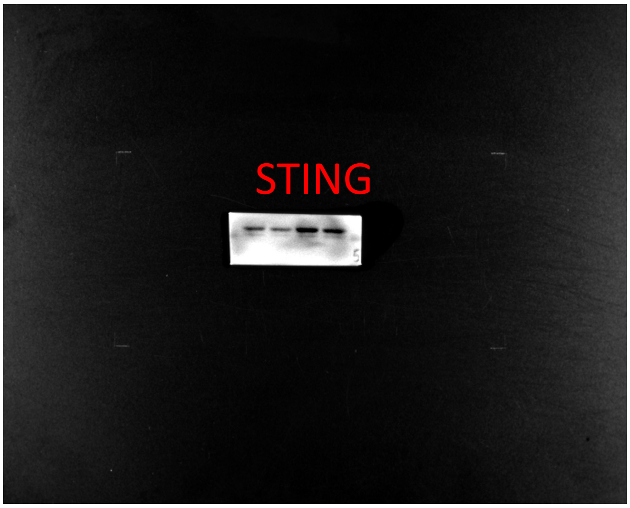

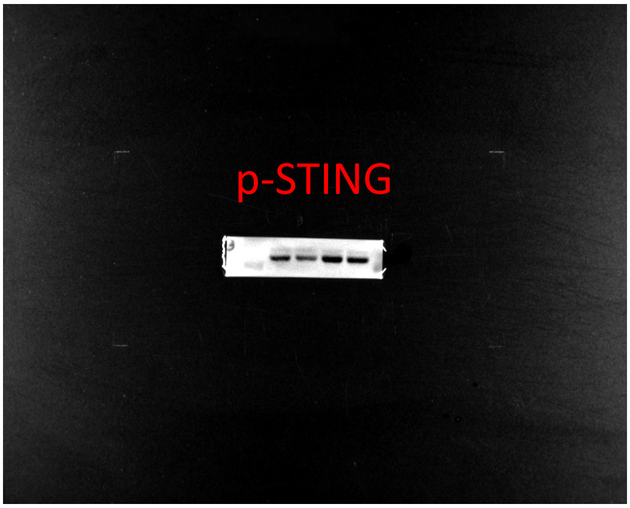

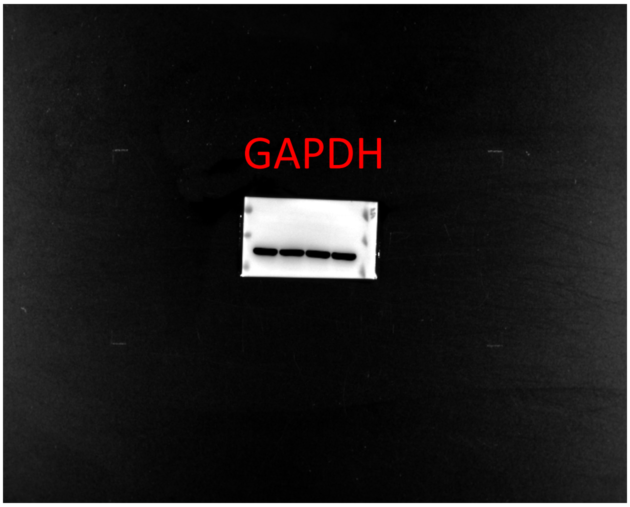

Figure 5. K

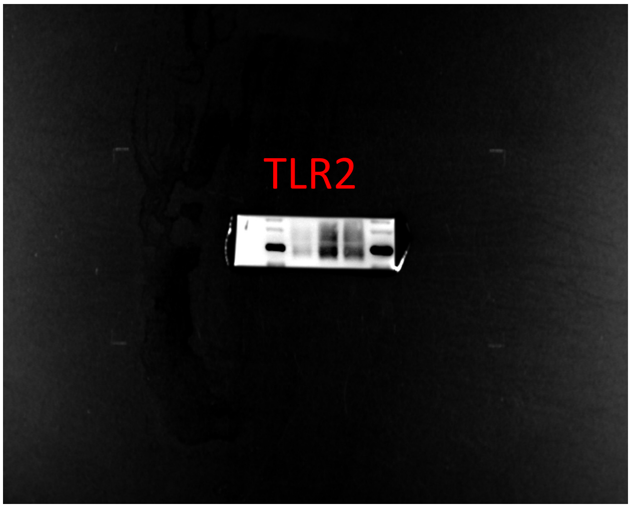

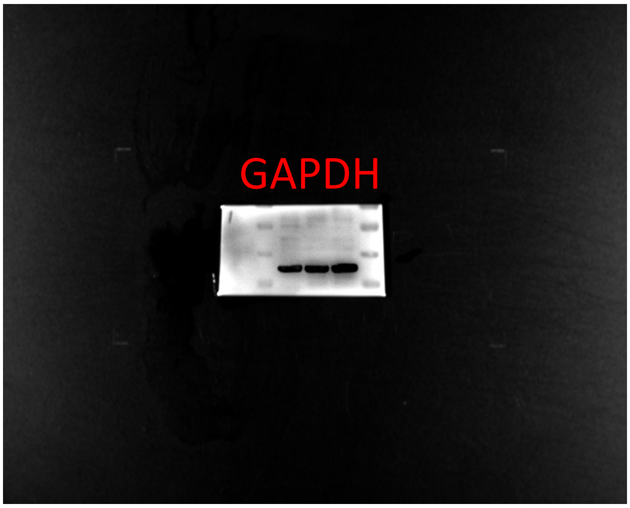

Figure 5. L

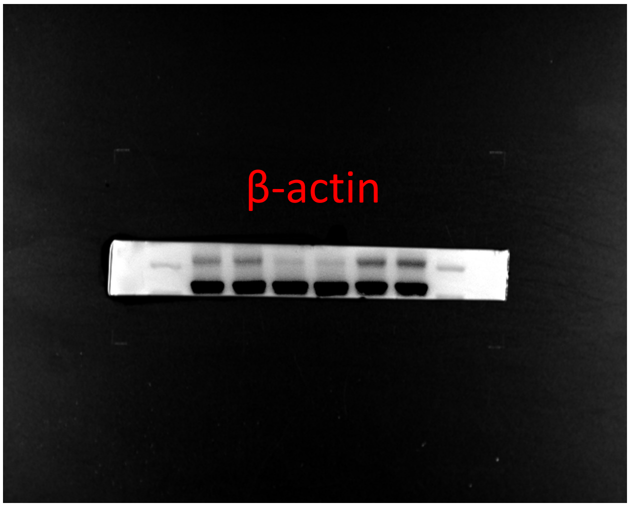

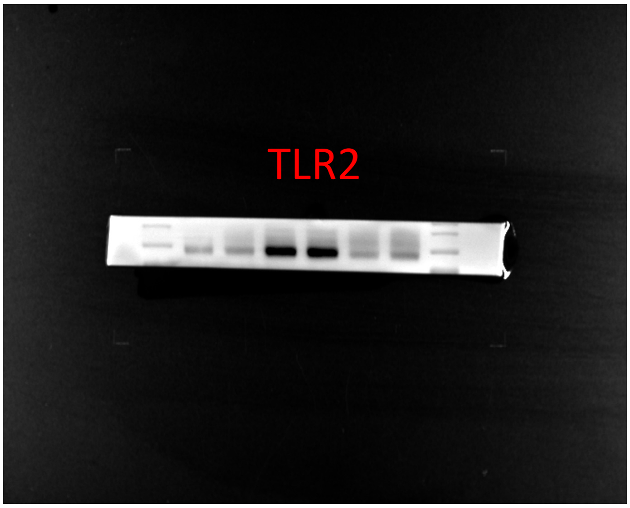

Figure 6. E

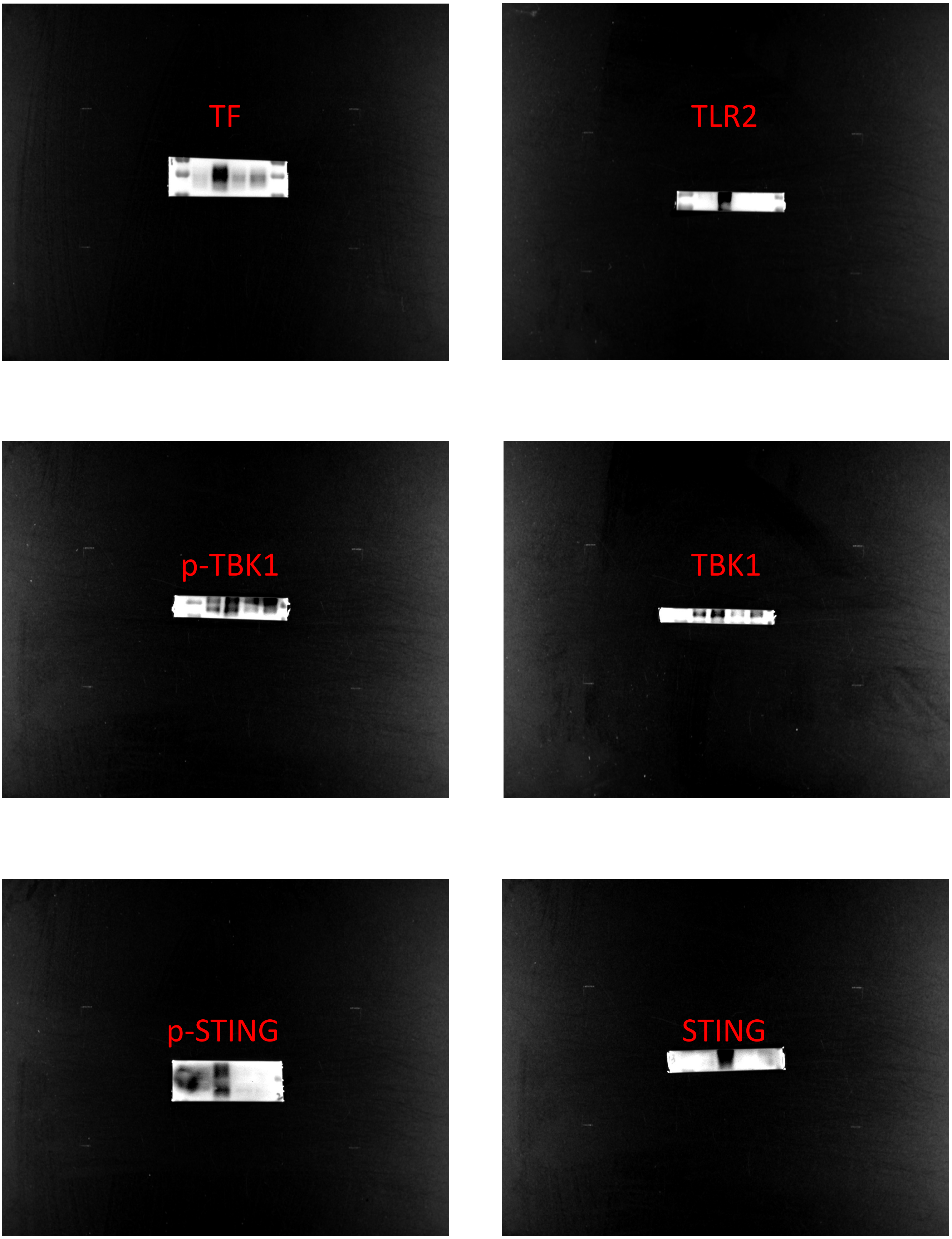

Figure 6. E-continued

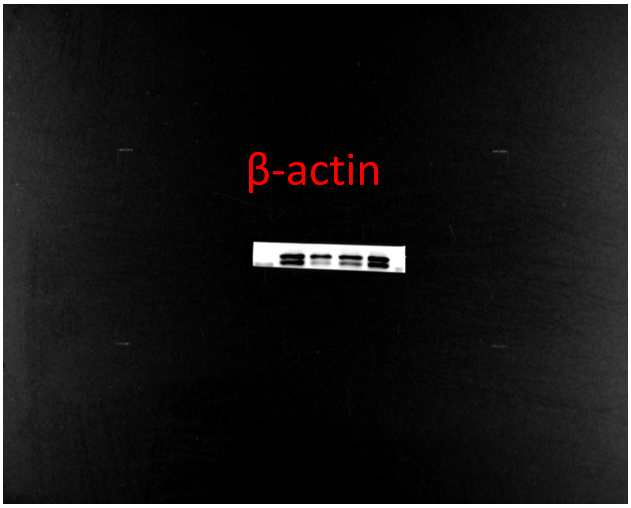

Figure 7. E

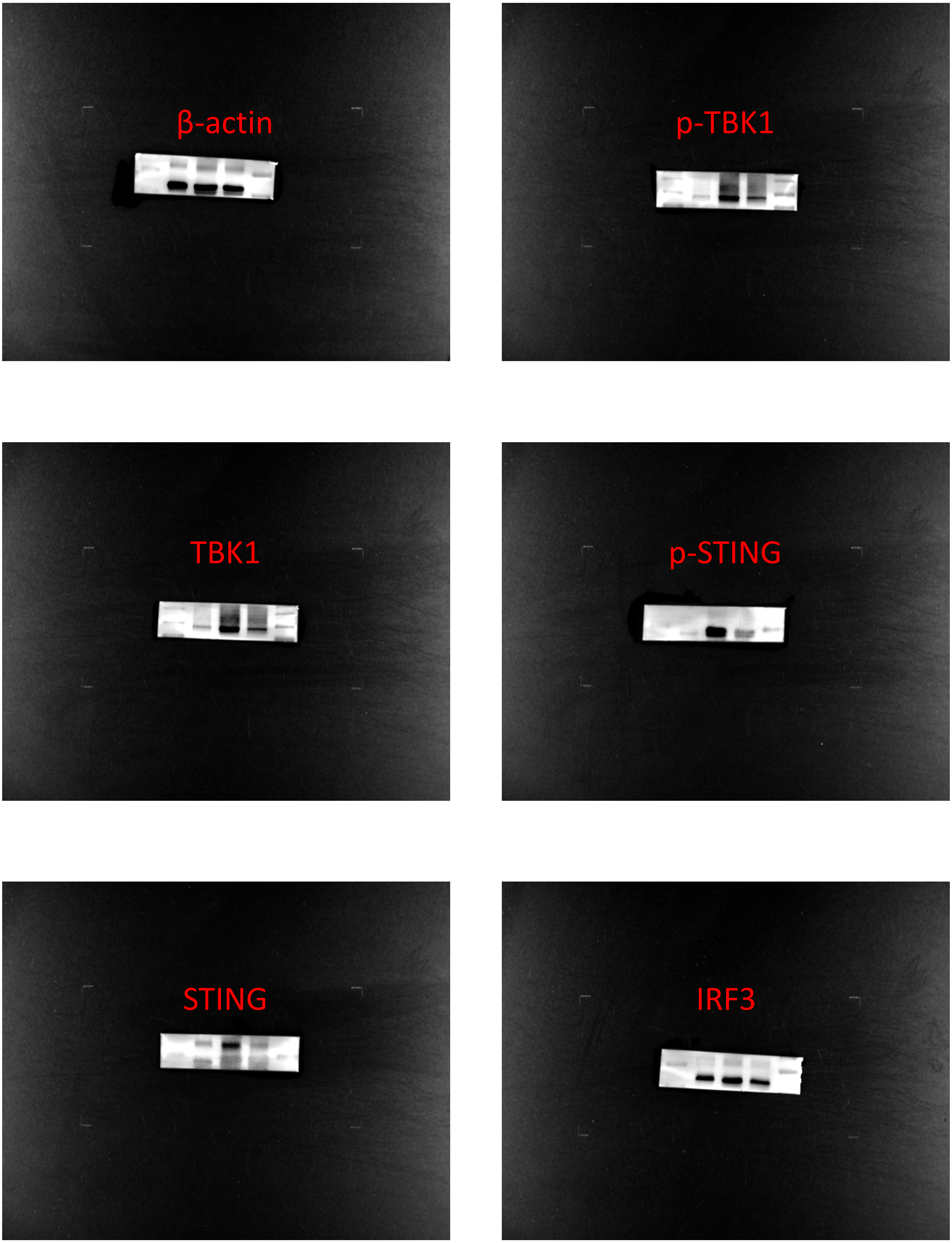

Figure 7. E-continued

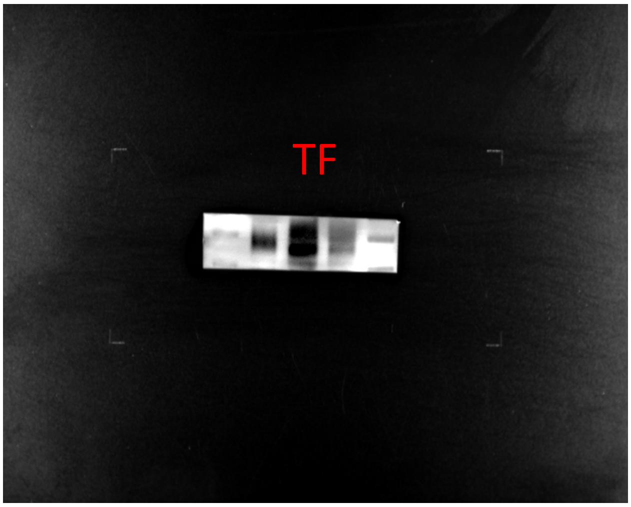

Figure 7. G

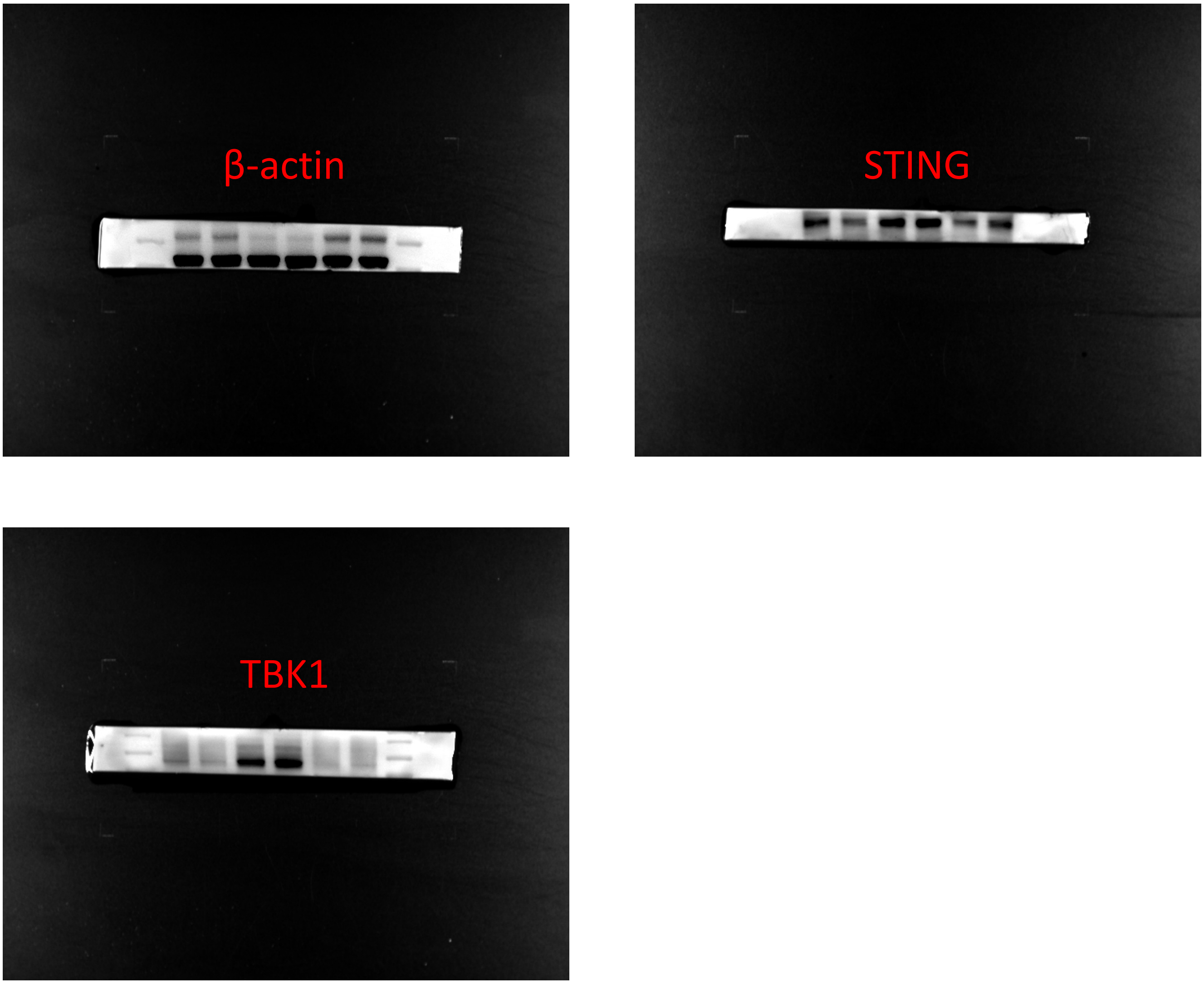

Supplement: Supplementary file 6 — Original Data File [file 41420_2023_1614_MOESM6_ESM.pdf]
